# Regulation of pericentromeric DNA loop size via Scc2-cohesin interaction

**DOI:** 10.1016/j.isci.2025.112322

**Published:** 2025-03-30

**Authors:** Sao Anh Nguyen, Toyonori Sakata, Katsuhiko Shirahige, Takashi Sutani

**Affiliations:** 1 Institute for Quantitative Biosciences, The University of Tokyo 1-1-1 Yayoi, Bunkyo-Ku, Tokyo 113-0032, Japan; 2 Department of Cell and Molecular Biology, Karolinska Institutet Tomtebodavägen 16, 171 77 Stockholm, Sweden

**Keywords:** Natural sciences, biological sciences, cell biology, functional aspects of cell biology

## Abstract

Cohesin exhibits DNA loop extrusion when bound to the ATPase activator Scc2 (NIPBL in humans), which has been proposed to organize higher-order chromosome folding. In budding yeast, most chromosome-bound cohesins lack Scc2. How the Scc2-cohesin interaction is regulated on the chromosome and its physiological consequences remain unclear. Here, we show that the deletion of both *ECO1* and *WPL1*, two known cohesin regulators, but not either alone, caused Scc2-cohesin co-localization in metaphase, particularly around centromeres, using calibrated chromatin immunoprecipitation sequencing (ChIP-seq). Eco1’s mitotic activity was required to prevent this co-localization in Δ*wpl1.* We also demonstrate that Scc2-cohesin co-localization enlarged pericentromeric DNA loops, linking centromeres to genome sites hundreds of kilobases away, and delayed mitotic chromosome segregation. These findings suggest that Wpl1 and Eco1 cooperatively regulate Scc2-cohesin interaction, restrict pericentromeric DNA loop size, and facilitate chromosome segregation.

## Highlights

• Scc2-cohesin interaction regulates higher-order chromosome folding in yeast
• *ECO1* and *WPL1* double deletion induce Scc2-cohesin co-localization in metaphase
• This co-localization enlarges pericentromeric DNA loops and delays segregation
• Eco1’s mitotic function is required to prevent Scc2-cohesin interaction

## Graphical abstract

**Figure:**
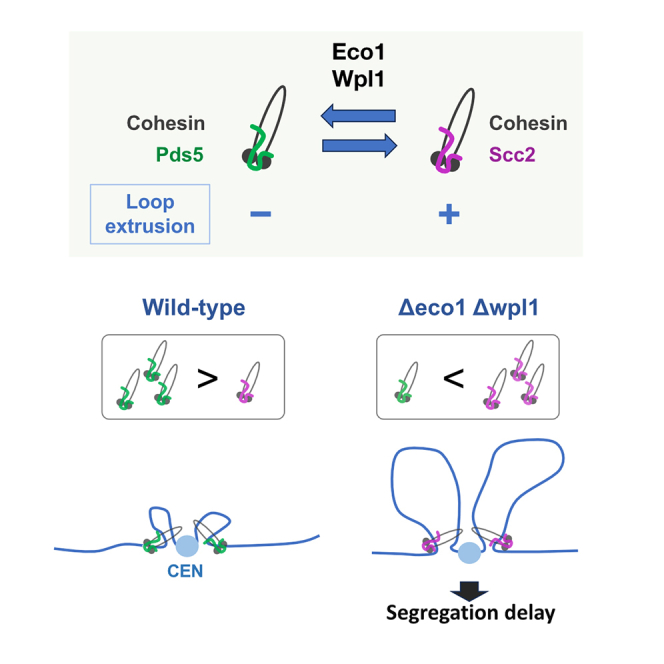

## Introduction

Recent developments in genome-wide chromosomal conformation capture techniques, such as the Hi-C method, have revealed the principle of higher-order chromosome folding. The formation of DNA loops and sub-Mb-scale self-interacting regions (i.e., regions within which DNA interacts with each other at high frequency) have been found in various species, including yeasts and mammals, and are thought to be a fundamental feature of chromosome structure.^1^^,^^2^^,^^3^ A prominent example of the self-interacting domains is the topologically associated domain (TAD) found in mammalian nuclei.^4^

Cohesin is one of the structural maintenance of chromosomes (SMC) protein complexes and plays a significant role in the formation of DNA loops and TAD structures.^1^^,^^3^^,^^5^ It is composed of four subunits, Smc1, Smc3, Scc1/Rad21, and Scc3 (SA1 or SA2 in humans), and exhibits an overall ring-like configuration. Recently, cohesin was shown to introduce loop structures to DNA in the presence of ATP and Scc2-Scc4 dimer, an activator of cohesin ATPase.^1^^,^^6^^,^^7^^,^^8^ NIPBL (Nipped-B-like), the human homolog of Scc2, is actually required for TAD formation.^9^^,^^10^ Scc2 directly interacts with cohesin at multiple interfaces.^11^ In the loop extrusion reaction, cohesin first forms a small loop at the DNA site it is bound to and then gradually increases the size of the loop by extruding DNA through its ring. Loop extrusion by cohesin can be impeded by various roadblocks on DNA. A typical example is the CTCF (CCCTC-binding factor) protein, whose binding sites are enriched at TAD boundaries.^12^^,^^13^^,^^14^^,^^15^^,^^16^ The structural features of the TAD observed by Hi-C are well explained by the model that the cohesin-mediated expansion of DNA loops starting at any point in the genome is restricted by the CTCF binding sites.^17^ In addition to TAD formation, cohesin-mediated loop extrusion is reportedly important for transcription control, DNA repair, and V(D)J recombination processes in mammals.^1^^,^^5^^,^^18^

Cohesin activity is regulated by various factors other than Scc2. Pds5 binds to cohesin in a mutually exclusive manner with Scc2, and the binding of Pds5 makes cohesin ATPase inactive.^19^^,^^20^^,^^21^ Wpl1 (WAPL) interacts with cohesin via Pds5 and induces cohesin dissociation from chromosomes.^22^^,^^23^^,^^24^^,^^25^^,^^26^ Before its function in loop extrusion was discovered, cohesin was known for its canonical role in sister chromatid cohesion.^27^ The establishment of sister chromatid cohesion requires acetylation of the Smc3 subunit by Eco1 (ESCO1 or ESCO2) in a manner coupled with DNA replication.^24^^,^^28^^,^^29^^,^^30^^,^^31^ Acetylation of cohesin is suggested to weaken its interaction with Scc2.^32^^,^^33^^,^^34^ The size of the cohesin-mediated loops is expected to vary depending on the affinity of cohesin for Scc2 and the retention time of cohesin on DNA. Indeed, in human cells lacking PDS5, WAPL, or ESCO1, the size of cohesin-mediated loops is larger, and loops are more easily formed to overcome barriers imposed by CTCF.^9^^,^^35^^,^^36^^,^^37^

Cohesin-dependent loops and self-interacting regions are also found in chromosomes of budding yeast *Saccharomyces cerevisiae*.^38^^,^^39^^,^^40^^,^^41^ CTCF is not present in this organism, and the boundaries of the self-interacting regions are defined by other means.^42^ Cohesin loop expansion is negatively regulated by Pds5, Wpl1, and Eco1 in yeast,^39^^,^^43^ indicating the regulation of DNA loop enlargement is highly conserved between yeast and mammals. In wild-type (WT) mitotic cells whose centromeres are dissociated from microtubules, another prominent chromosome structure has been reported; the flanking regions adjacent to the centromere are linked with nearby cohesin binding sites to form so-called pericentromeric loops.^44^ These pericentromeric DNA loops are generated presumably by cohesin loaded at the core centromeres that are unattached to the mitotic spindle. The expansion of the loops is confined by the genes that are 10–30 kb away from and transcribed toward the centromeres, and this confinement ensures the maintenance of sister chromatid cohesion in regions distal to the centromeres. Reorientation of the border-defining genes resulted in the loss of cohesion in a broader genome region and impairs the establishment of chromosome biorientation under the spindle tension.^44^

A remarked difference in the behavior of budding yeast cohesin from that of mammalian one is that the vast majority of cohesin on the genome is not bound to Scc2/NIPBL^45^^,^^46^ and presumably exhibits only limited ATPase activity. This may be related to the relatively small size of the DNA loops in this organism.^40^ It remains to be understood how the Scc2-cohesin interaction is controlled on budding yeast chromosomes and what physiological consequences malfunction in the control mechanism causes. Here, we revealed that simultaneous deletion of Wpl1 and Eco1 resulted in co-localization of Scc2 with cohesin specifically on mitotic chromosomes without spindle tension. The Δ*wpl1* Δ*eco1* cells also exhibited extensively enlarged pericentromeric DNA loops, whose anchor sites corresponded to the co-localization sites of Scc2 and cohesin. The formation of the enlarged loops depended on the presence of Scc2 in mitosis, and Wpl1 and Eco1 synergistically contributed to the loop size restriction. Finally, we found that Δ*wpl1* Δ*eco1* cells showed delayed progression of chromosome segregation and that this phenotype was rescued by acute depletion of Scc2 in mitosis. Collectively, the data demonstrate that Wpl1 and Eco1 cooperatively control Scc2-cohesin interaction, restrict the size of the pericentromeric DNA loops, and facilitate unperturbed chromosome segregation.

## Results

### Simultaneous depletion of Wpl1 and Eco1 promoted Scc2 co-localization at the cohesin binding sites

**Figure 1:**
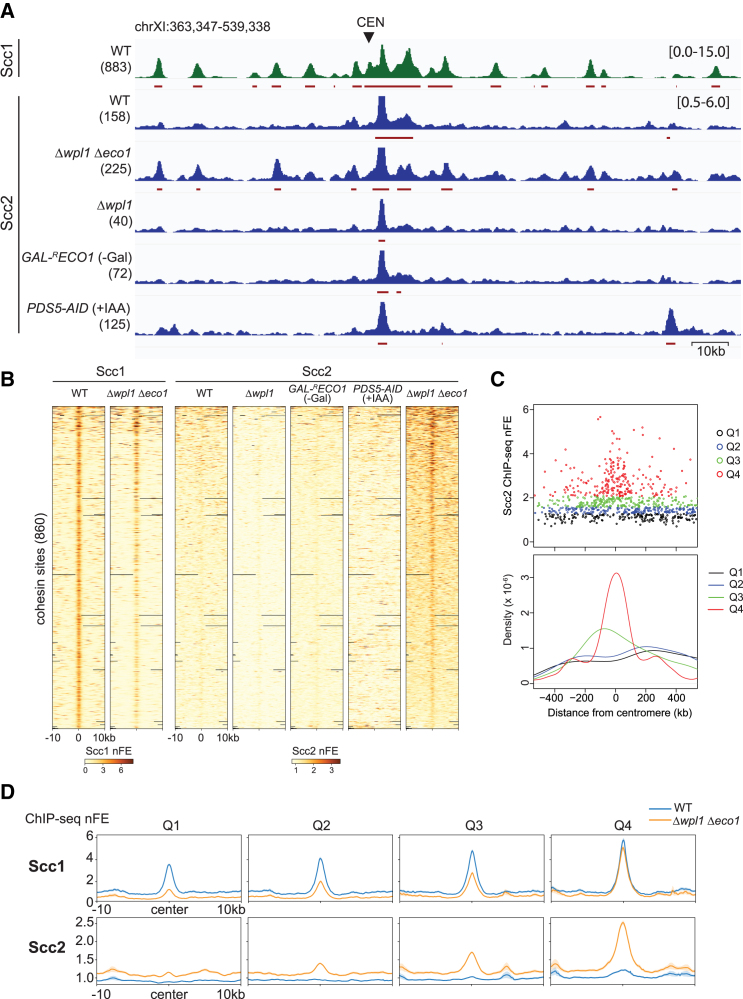
Simultaneous depletion of Wpl1 and Eco1 promotes Scc2 co-localization at cohesin binding sites (A) Calibrated ChIP-seq profiles of Scc1-PK in wild-type (WT, strain SN75) and Scc2-PK in WT (SN40), Δ*wpl1* Δ*eco1* (SN54), *Δwpl1* (SN53), *GAL-*^*R*^*ECO1* (SN41) in galactose-free medium, and *PDS5-AID* (SN80) treated with IAA. Cells were arrested at metaphase by benomyl treatment. See Figure S1 for details on the culture conditions of *GAL-*^*R*^*ECO1* and *PDS5-AID*. The y axis indicates normalized fold enrichment (nFE), or ChIP/input ratio normalized by spike-in control, with the range in brackets. CEN, centromere. The identified peaks are indicated by red horizontal bars, and the number of the peaks across the genome is shown in parentheses. (B) Heatmap of Scc1 and Scc2 ChIP-seq nFE in the indicated strains. 10kb-surrounding regions of the cohesin binding sites in WT (excluding those less than 5 kb to the centromeres) are depicted. Regions are sorted in descending order of Scc2 nFE in Δ*wpl1* Δ*eco1*. In addition to the aforementioned strains, Δ*wpl1* Δ*eco1* with *SCC1*-PK gene (SN74) was used. (C) (Top) The scatterplot showing Scc2 nFE and the distance from the centromeres at each non-centromeric cohesin binding site in WT. Overlay representation of all chromosomes. The cohesin sites are divided into four groups according to their Scc2 FE and depicted by different colors. (Bottom) The kernel density estimation plot for the cohesin sites in each group. (D) Aggregated ChIP-seq profiles of Scc1 and Scc2 in WT and Δ*wpl1* Δ*eco1* for each group of the cohesin sites. The profiles are centered at the summit of the Scc1 peaks. Bold line, mean; shaded area, 95% confidence interval. See also Figures S1–S5.

We explored how the genome-wide localization of Scc2, the cohesin ATPase activator, was affected by mutations in the cohesin regulators by calibrated chromatin immunoprecipitation sequencing (ChIP-seq). First, we analyzed the localization in the cells arrested in metaphase by benomyl, a microtubule-destabilizing drug. Proper arrest was verified by flow cytometry analysis (Figure S1). Chromosomal binding of Scc2 was displayed using normalized fold-enrichment (nFE) or ChIP/input ratio after normalization by spike-in control. In WT metaphase cells, centromeres and the surrounding regions were the major Scc2 binding sites (Figure 1A). On chromosome arms, Scc2 binding sites were coincident with those of Rpo21, a subunit of RNA polymerase II, most of which were not occupied by the cohesin subunit Scc1 (Figures 1A, S2A, and S2B). These binding sites are identical to those previously reported and assumed to correspond to the cohesin loading sites.^45^^,^^47^ We found that double deletion of *WPL1* and *ECO1* (Δ*wpl1* Δ*eco1*) resulted in novel, prominent peaks of Scc2 along chromosome arms in metaphase (Figure 1A). Note that *ECO1* is an essential gene, but Δ*eco1* becomes viable by simultaneous deletion of *WPL1* gene.^26^^,^^28^ The number of Scc2 peaks in the genome was 225, 42% more than that for WT (Figure S2B). Notably, the majority (67%) of Scc2 peaks in Δ*wpl1* Δ*eco1* overlapped with Scc1’s peaks (Figure S2B). Co-localization of Scc1 and Scc2 in Δ*wpl1* Δ*eco1* was confirmed by correlation analysis; Spearman’s correlation coefficient increased from 0.16 in WT to 0.49 in Δ*wpl1* Δ*eco1* (Figure S2C). Calibrated ChIP-seq of Scc1 and Scc2 showed a high correlation between two biological replications, both for WT and Δ*wpl1* Δ*eco1* (Figure S3A).

The co-localization of Scc2 with Scc1 required simultaneous deletion of *WPL1* and *ECO1* (Figure 1A). It was not observed in Δ*wpl1* or the cells in which Eco1 was depleted by promoter shut-off (*GAL-*^*R*^*ECO1*)^48^ (Figures 1A and S4). In the Eco1 depletion experiment, cells were allowed to progress through S phase in the absence of Eco1 and arrested in metaphase (Figure S1C). Scc2 co-localization was not observed either in the metaphase cells in which Pds5 was acutely degraded by the auxin-inducible degron (AID) system (*PDS5-AID*) (Figures 1A and S1B). We compared the genome-wide binding profile of Scc1 and Scc2. Heatmap showing the nFE value around all cohesin binding sites on chromosome arms revealed that most of the cohesin sites were co-occupied with little, if any, Scc2 in WT, Δ*wpl1*, *GAL-*^*R*^*ECO1*, and *PDS5-AID* (Figure 1B). In contrast, Scc2 was found to accumulate at a majority of the same cohesin sites in Δ*wpl1 Δeco1*, although its binding intensity was not sufficient to be identified as a peak by the peak-calling algorithm at most sites (Figure 1B). ChIP-qPCR also confirmed the co-localization of Scc2 at the cohesin sites exclusively in Δ*wpl1* Δ*eco1* (Figure S3B).

We noticed that the intensity of Scc2 binding at the cohesin sites in Δ*wpl1* Δ*eco1* varied from site to site. To elucidate the nature of the strong Scc2 binding sites, the cohesin sites were equally divided into four groups based on Scc2 ChIP-seq nFE at each site (Q1–Q4 in order of increasing nFE). The cohesin sites with the highest Scc2 binding (group Q4) were found to be enriched in regions less than 100 kb from the centromeres (Figure 1C). As mentioned previously, Δ*wpl1* Δ*eco1* mutation caused a reduction of Scc1 ChIP-seq peak height. The ChIP-seq profiles averaged over the cohesin sites in each group indicated that the degree of the reduction in Scc1 binding was more severe in groups Q1 and Q2; Scc2 and Scc1 binding intensities were, hence, exhibited a high correlation in Δ*wpl1* Δ*eco1* (Figure 1D). The sites with intense Scc2 binding were also correlated well with those showing strong interaction with the centromere through DNA loops (see in the following text).

Pds5 is a mutually exclusive antagonist of Scc2 in association with cohesin.^19^^,^^20^ We investigated the chromosomal binding of Pds5 in Δ*wpl1* Δ*eco1* strain by calibrated ChIP-seq and ChIP-qPCR. The results consistently showed that Pds5 binding at the cohesin sites was significantly decreased in Δ*wpl1* Δ*eco1* strain (Figure S5), further verifying the increase of Scc2 binding at these sites. Unlike cohesin sites on chromosome arms, the amount of Pds5 bound to the centromeres increased in Δ*wpl1* Δ*eco1* cells (Figure S5B). This phenomenon has been reported previously.^49^ The contrasting behavior of Pds5 at centromeres and cohesin sites is likely also present in Δ*wpl1* single mutant (Figure S5C).^49^ It remains unknown why the effect of Δ*wpl1* Δ*eco1* mutation on Pds5 chromosomal binding is different between the centromeres and chromosome arms.

**Figure 2:**
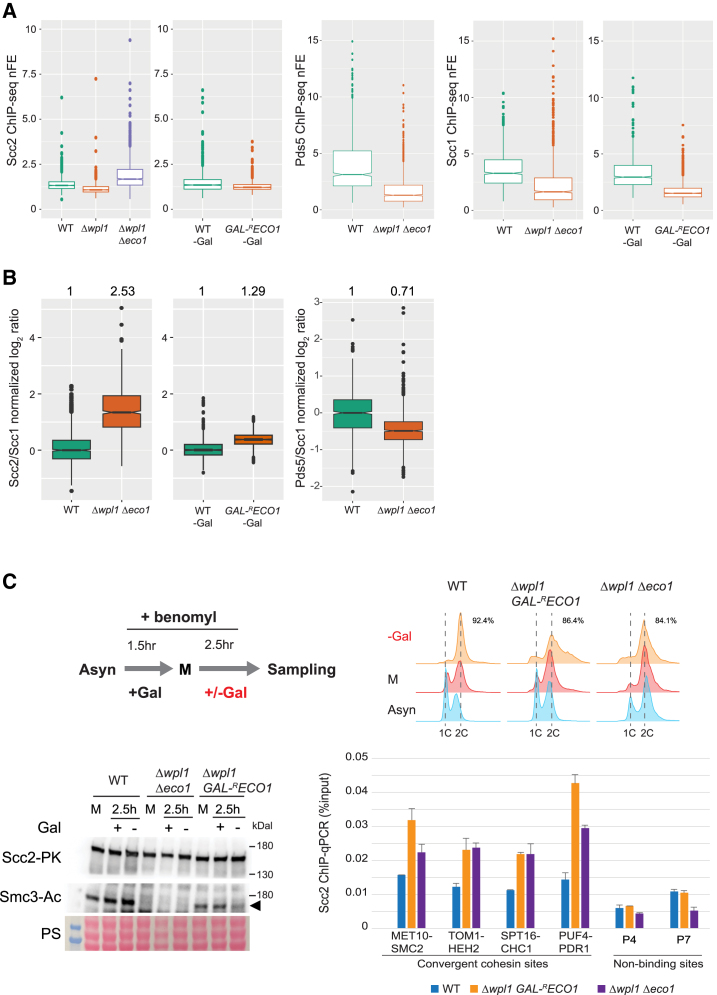
The deletion of Wpl1 and Eco1 synergistically enhances the interaction between Scc2 and cohesin during metaphase (A) Boxplots to compare Scc2, Pds5, and Scc1 ChIP-seq nFE values at the cohesin binding sites in indicated conditions. (B) Boxplots to compare the ratio of Scc2's nFE to Scc1’s nFE, or Pds5’s nFE to Scc1’s nFE at the cohesin sites in indicated conditions (in log_2_ scale). The ratios were normalized by setting the median for WT to 1. The numbers at the top of the plots represent the median in linear scale. (C) (Top) Schematic representation of the experimental protocol to deplete Eco1 after metaphase arrest and cell-cycle arrest monitored by flow cytometry (numbers indicate the proportion of cells with 2C DNA content). WT (SN40), Δ*wpl1 GAL-*^*R*^*ECO1* (SN722), and Δ*wpl1* Δ*eco1* (SN54) strains with *SCC2*-PK gene were used. Asyn, asynchronous; M, metaphase; Gal, galactose. (Bottom left) Western blot assessing Smc3 acetylation level (Smc3-Ac). PS, Ponceau S staining as a loading control. (Bottom right) ChIP-qPCR of Scc2 in the indicated conditions. Analyzed loci are four representative cohesin binding sites and two non-binding sites. The mean of two technical replications was shown. Error bar, standard deviation. See also Figures S6–S10.

In Δ*wpl1* Δ*eco1* mutant and Eco1-depleted cells, the amount of cohesin on chromosomes was reduced, though the binding location was unchanged (Figures 1B, 2A and S4). To quantitatively evaluate how Δ*wpl1* Δ*eco1* mutation or Eco1-depletion alters the affinity between cohesin and Scc2, we calculated the ratio of Scc2 to Scc1 nFE values at cohesin binding sites on chromosomes (Figure 2B). Δ*wpl1* Δ*eco1* mutation markedly increased the Scc2/Scc1 ratio (2.53-fold), indicating an enhanced affinity of Scc2 for Scc1. This increase was consistently observed throughout the genome (Figure S6). A corresponding decrease in the affinity of Pds5 for Scc1 was also observed in Δ*wpl1* Δ*eco1* mutant (Figure 2B). In contrast, cells lacking only Eco1 displayed a much weaker increase (1.29-fold) in the Scc2/Scc1 ratio. In *Δwpl1* mutant, the amount of Scc2 bound to the cohesin sites was reduced to ∼80% (Figure 2A). Taking into consideration the fact that Scc1 on chromosomes is reduced to ∼55% in *Δwpl1*,^26^ Wpl1 deletion presumably increases the Scc2-cohesin interaction slightly (∼1.5-fold). These results indicate that the deletion of Eco1 and Wpl1 synergistically enhances the affinity of Scc2 for cohesin.

### Scc2-cohesin co-localization is specific to metaphase and impeded by mitotic function of Eco1

We conducted Scc1 and Scc2 ChIP-seq in S phase, where the establishment of sister chromatid cohesin was in progress. The cells were arrested in G1 phase by α factor, then released into an α factor-free medium for 30 min to synchronize them in S phase (Figure S7A). Scc1 ChIP-seq profiles were similar between S and metaphase, though the binding was less intense, and the number of the detected peaks was decreased in S phase (Figures S7B and S7C). In WT cells, Scc2 binding profiles were similar between S and metaphase, with the only difference being that the binding at the centromere regions was less prominent in S phase (Figure S7C). Contrarily, in Δ*wpl1* Δ*eco1* cells, the co-localization of Scc2 at the cohesin sites was observed only in metaphase; the profile in S phase was almost identical to that in WT, and no increase in Scc2 binding at the cohesin sites was observed (Figures S7C and S7D). Hence, the co-localization of Scc1 and Scc2 in Δ*wpl1* Δ*eco1* was specific to metaphase.

The presence or absence of spindle tension affects the behavior of cohesin around the centromeres.^44^^,^^50^ We conducted Scc2 ChIP-qPCR in Δ*wpl1* Δ*eco1* cells arrested in metaphase by Cdc20 depletion, in which the centromeres were under tension (Figure S8). Unlike benomyl-treated metaphase cells, the Scc2 binding level at the cohesin sites was comparable to that in wt (Figure S8). The absence of spindle tension is presumed to enhance the co-localization of Scc2 and cohesin in Δ*wpl1* Δ*eco1*.

The essential role of Eco1 is to establish sister chromatid cohesion in the S phase. Next, we examined whether Eco1 should function outside the S phase to impede the Scc2-cohesin co-localization. Δ*wpl1 GAL-*^*R*^*ECO1* cells were first arrested in metaphase by benomyl, then transferred to a galactose-free medium and cultured for 2.5 h to deplete Eco1 protein specifically in metaphase (Figures 2C and S9). Smc3 acetylation level was reduced by about half compared with the cells cultured in galactose-containing medium, indicating that Eco1 continuously acetylates Smc3 in benomyl-induced metaphase cells. Scc2 ChIP-qPCR revealed that mitotic depletion of Eco1 in Δ*wpl1* was sufficient to elevate Scc2 binding at the cohesin sites to a comparable level as Δ*wpl1* Δ*eco1* (Figures 2C and S9). We also conducted an experiment where Δ*wpl1 GAL-*^*R*^*ECO1* cells were arrested at metaphase in a galactose-free medium and subsequently induced to express Eco1 (Figure S10). Upon induction, Scc2 binding to cohesin sites was decreased while Smc3 acetylation level was increased. Therefore, we conclude that Eco1 plays a role in counteracting the Scc2-cohesin interaction in mitotic cells without spindle tension. Unless otherwise stated, benomyl was used to induce metaphase arrest in the following experiments.

### Δ*wpl1* Δ*eco1* promotes the extension of pericentromeric DNA loops

**Figure 3:**
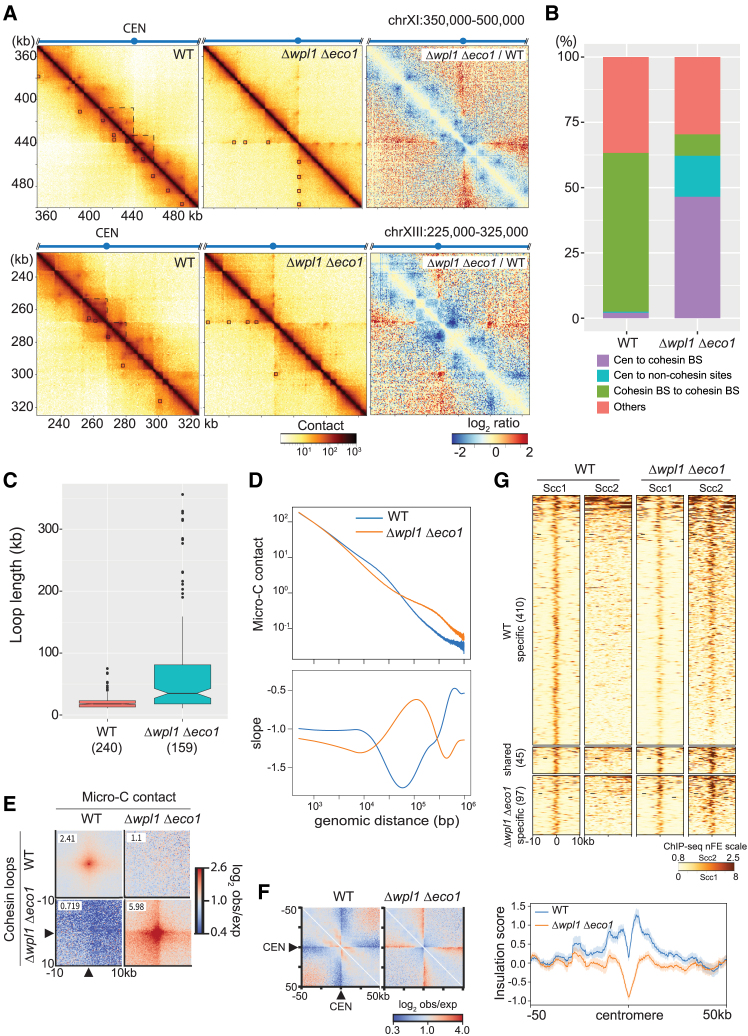
Δ*wpl1* Δ*eco1* promotes extension of pericentromeric DNA loops (A) Micro-C contact maps in WT (SKY001), Δ*wpl1* Δ*eco1* (KT110), and log2-ratio between Δ*wpl1* Δ*eco1* and WT. Cells were arrested at metaphase by benomyl treatment. Bin size is 500 bp. Squares in the contact maps indicated loops called by HICCUPs. Dashed lines mark the chromosomal domains adjacent to centromeres. Two representative regions in chromosomes XI and XIII are shown. The number of valid pairs in each sample was normalized to 115 million. CEN, centromere. (B) Proportion of the detected loops in each indicated category. Cen, centromere; cohesin BS, cohesin binding site. (C) Length distribution of cohesin loops (loops connecting between cohesin BSs) in WT and Δ*wpl1* Δ*eco1*. (D) Contact-versus-distance decaying curves of the Micro-C contact matrix in WT and Δ*wpl1* Δ*eco1*, and their first derivatives (slope). (E) Average contact frequency between cohesin loop anchors in WT and Δ*wpl1* Δ*eco1*. Observed/expected ratio of the contact frequency around the off-diagonal peaks connecting two cohesin loop anchors found in WT or Δ*wpl1* Δ*eco1* were averaged and plotted. The number in the top-left corner of each plot indicates the average enrichment score of the 3 × 3 central pixels. (F) (Left) Average contact frequency around the centromeres in WT and Δ*wpl1* Δ*eco1*. Black triangles indicate the centromere position. (Right) Averaged insulation score around the centromeres in WT and Δ*wpl1* Δ*eco1*. Bold line, mean; shaded area, 95% confidence interval. (G) Heatmaps of Scc1 and Scc2 ChIP-seq nFE in WT and Δ*wpl1* Δ*eco1*. 10-kb surrounding regions of the cohesin-bound loop anchors specific to WT, specific to Δ*wpl1* Δ*eco1*, and shared between WT and Δ*wpl1* Δ*eco1* are depicted. The number of sites for each group is shown in parentheses. See also Figures S11 and S12.

Since Scc2 binding confers the loop extrusion activity to cohesin, we investigated genome DNA folding of Δ*wpl1* Δ*eco1* cells using Micro-C technique. Micro-C, an improved version of Hi-C, can reveal chromosome folding with higher resolution^40^ and indeed enabled us to detect each loop clearly without the need for “pile-up” analysis in yeast chromosomes. In WT, consistent with previous study,^38^ short DNA loops of several tens of kb, which connect adjacent cohesin binding sites, were observed along the entire length of the chromosomes (Figures 3A and 3B). These short DNA loops largely disappeared in Δ*wpl1* Δ*eco1* mutant. A similar disappearance of DNA loops along chromosome arms was described in Pds5*-*depleted cells previously.^38^^,^^43^ The pericentromeric loops in WT, which are around 10 kb in length, were also greatly diminished in Δ*wpl1* Δ*eco1* (Figures 3A and S11). In contrast, we observed novel, longer DNA loops connecting a centromeric region and specific sites on the same chromosome in Δ*wpl1* Δ*eco1* (Figures 3A and 3B). The loops detected in Δ*wpl1* Δ*eco1* were smaller in number (159 compared with 240 in WT), but longer in length (mean loop length of 67 kb in Δ*wpl1* Δ*eco1* compared with 20 kb in WT) (Figure 3C). Consistently, the contact-versus-distance decaying curve and its derivative (Figure 3D) confirmed a relative decrease in short-range contacts (<50 kb) and a relative increase in long-range contacts (>50 kb). A similar promotion of long-range interactions in cells lacking both Wpl1 and Eco1 was reported previously.^39^ In addition to loop length, the loop interaction was also more intense in Δ*wpl1* Δ*eco1* mutant than in WT cells, as revealed by the aggregate peak analysis (APA) plots over the cohesin-positive loops (Figure 3E).

Δ*wpl1* Δ*eco1* also caused changes in the chromatin compartmentalization around centromeres in Δ*wpl1* Δ*eco1*, as shown in Figure 3A. First, self-interactions within a region approximately 10–20 kb in size and adjacent to the centromere, which is prominent in WT, became weaker in Δ*wpl1* Δ*eco1*. Second, the centromere-adjacent regions on both chromosome arms became more insulated in Δ*wpl1* Δ*eco1*. These points were verified by the normalized APA plot of contact frequency on all centromeres (Figure 3F, left). The averaged insulation score profiles around the centromeres (Figure 3F, right) also confirmed the disappearance of inter-arm interactions in Δ*wpl1* Δ*eco1*; a decreased insulation score at the centromere indicates less-than-expected interactions across it. Together, the data indicate that Δ*wpl1* Δ*eco1* caused drastic changes in higher-order chromosome folding, including the formation of larger pericentromeric loops.

### Novel loop anchors in Δ*wpl1* Δ*eco1* are associated with cohesin and Scc2 binding

As shown in Figure 3B, most of the DNA loops were anchored at cohesin binding sites. The number of the loop anchor sites with cohesin binding (excluding the centromere regions) was 455 and 142 in WT and Δ*wpl1* Δ*eco1*, respectively, and 45 of them were shared. We examined the binding of Scc2 at these sites by plotting the heatmaps of Scc2 ChIP-seq profile in a 10-kb flanking region centered at the cohesin-bound loop anchors (Figure 3G). In WT, Scc2 was hardly found at cohesin-bound loop anchors. Contrarily, in Δ*wpl1* Δ*eco1* cells, Scc2 binding was observed at the anchor sites. The binding was more intense in general at the anchors detected in Δ*wpl1* Δ*eco1*. Note that the WT-specific anchors also showed weak interactions with the centromeres specifically in Δ*wpl1* Δ*eco1* strain, which were below than the threshold for loop calling (Figure S12). These data suggest that the co-localization of cohesin and Scc2 is coupled with the emergence of novel DNA loops in Δ*wpl1* Δ*eco1.*

### DNA loop extension is blocked by centromere-oriented long genes

**Figure 4:**
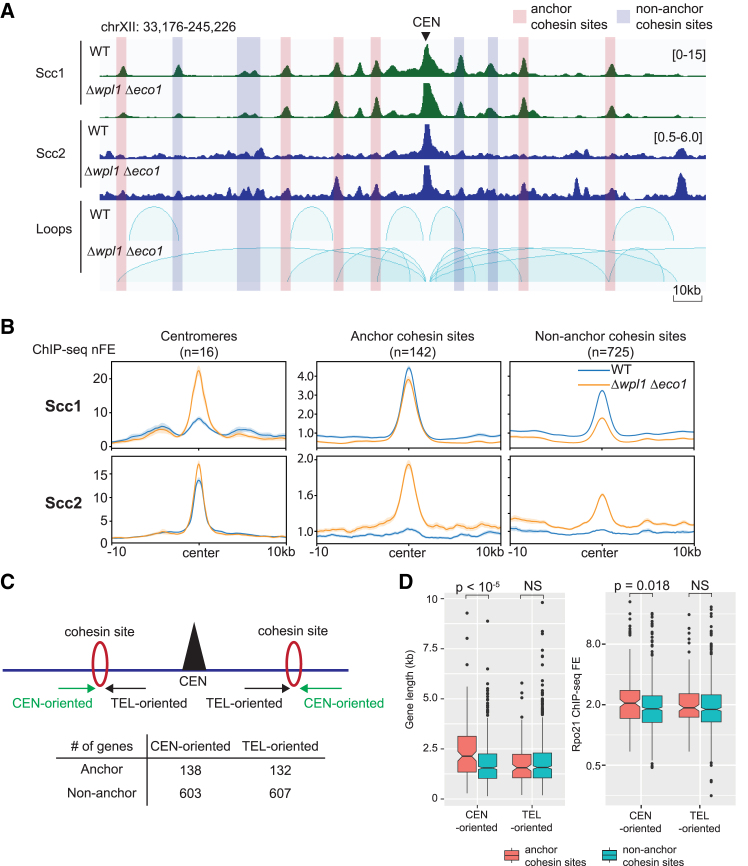
DNA loop expansion in Δ*wpl1* Δ*eco1* is impeded by long centromere-oriented genes (A) ChIP-seq profile of Scc1 and Scc2 alongside loops called by HICCUP in WT and Δ*wpl1* Δ*eco1* arrested at metaphase. The cohesin sites are categorized into anchor or non-anchor sites depending on their overlapping with anchors of centromere-originated loops in Δ*wpl1* Δ*eco1*. (B) Aggregated plots of Scc1 and Scc2 ChIP-seq nFE in WT and Δ*wpl1* Δ*eco1*. 10-kb surrounding regions around the centromeres, anchor cohesin sites, and non-anchor cohesin sites are depicted. Bold line, mean; shaded area, 95% confidence interval. (C) Labeling of the genes adjacent to the cohesin binding sites. CEN-oriented genes, genes distal to the centromere (CEN) and transcribed toward it. TEL-oriented genes, genes proximal to CEN and transcribed away from it. Note that most of the cohesin sites are located in convergent intergenic regions. The number of genes in each category is shown in the table. (D) Boxplot comparison of gene length and Rpo21 ChIP-seq FE between CEN- and TEL-oriented genes. The numbers above the plots are *p* values (Mann-Whitney U test, two-tailed). NS, non-significant difference (*p* > 0.05). See also Figures S13–S15.

The centromere-originated loops in Δ*wpl1* Δ*eco1* extended to the regions more than 100 kb from the centromere. This contrasts sharply with WT cells, where loops extend only a few tens of kb from the centromeres. As mentioned previously, these loops typically ended at the cohesin binding sites, but only a fraction of these sites served as the anchors. To clarify the nature of the anchor sites, we divided the cohesin binding sites in the genome into two groups: anchor (142) and non-anchor (813) cohesin sites, which exhibited or did not exhibit the interactions with the centromere on the same chromosome, respectively (Figure 4A). We found that the binding of both cohesin and Scc2 was more intense at the anchor cohesin sites, compared with the non-anchor cohesin sites (Figure 4B). Consistently, when the cohesin binding sites were grouped based on the level of co-localized Scc2 as in Figure 1D, the contact frequency between these sites and the centromere became more intense for the cohesin sites with higher Scc2 binding (Figure S13). We also noticed that Δ*wpl1* Δ*eco1* resulted in a substantial increase in cohesin binding at the centromeres (Figure 4B).

Gene transcription can be an obstacle to loop expansion.^42^^,^^44^^,^^51^ Thus, we addressed whether the neighboring genes of the anchor cohesin sites display any specific characteristics. As reported in Lengronne et al.*,*^47^ most cohesin binding sites in yeast were located in intergenic regions (IGRs) between two convergently oriented genes. We observed that genes located on the distal side to the centromere and transcribed toward the centromere (referred to as CEN-oriented genes, as illustrated in Figure 4C) were longer when associated with anchor cohesin sites compared to non-anchor cohesin sites (Figure 4D, left). Additionally, the transcriptional activity of these CEN-oriented genes, measured by Rpo21 ChIP-seq FE, was significantly higher at anchor cohesin sites (Figure 4D, right). In contrast, genes positioned proximal to the centromere and transcribed toward the telomeres (referred to as TEL-oriented genes, Figure 4C) did not exhibit any differences in either length or transcriptional activity between anchor and non-anchor cohesin sites (Figure 4D). We further confirmed no differences in the length or transcriptional activity of CEN- and TEL-oriented genes, when targeting all genes in the genome or the genes adjacent to the cohesin sites (Figure S14). These data imply that length and transcriptional activity of CEN-oriented genes play a substantial role in determining loop location in *Δwpl1 Δeco1.*

Besides loops extruded from centromeres, we also occasionally observed the “stripes” projecting from several loci on chromosome arms in Δ*wpl1* Δ*eco1* (Figure S15, left). Examining those loci revealed that the neighboring genes are either exceptionally long (e.g., *GCN1*, *MYO1*, and *FAS1*) or strongly transcribed (e.g., *PDR5*) (Figure S15, right). The loop anchors were coincident with the 3′ end of these genes and associated with high levels of cohesin binding. The loops consistently extend only toward the downstream of the genes. We speculate that these genes act as strong, direction-dependent barriers to the expansion of loops initiated at some point downstream of the gene, thereby resulting in the stripe pattern in the Micro-C contact map.

### Synergistic effect of Δ*wpl1* and *Δeco1* in loop extension

**Figure 5:**
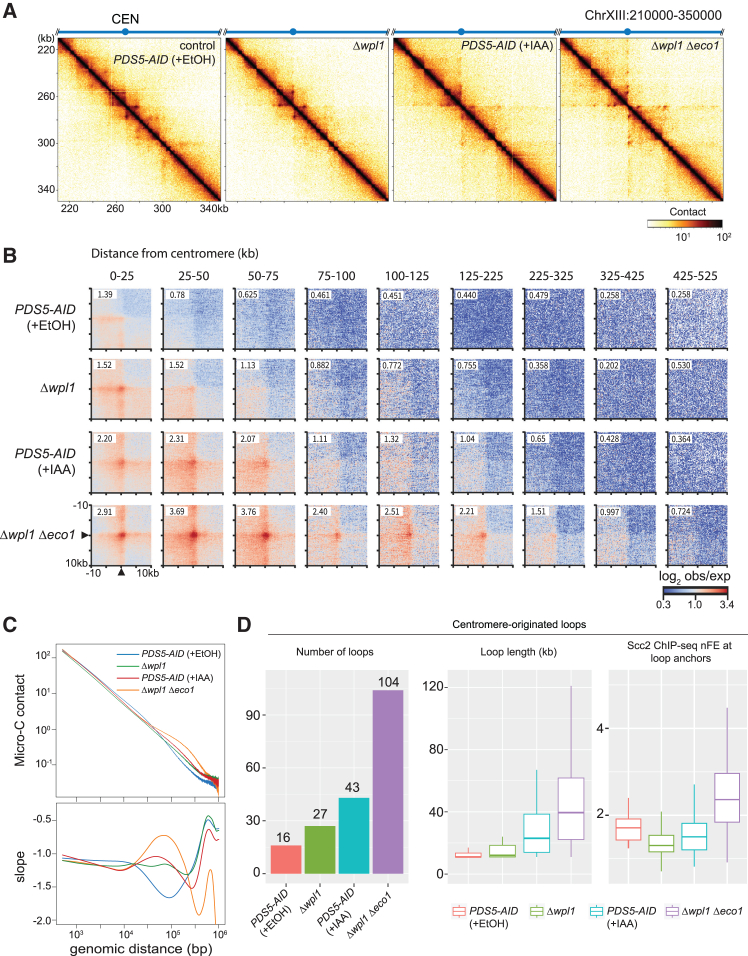
Synergetic effect of Wpl1 and Eco1 depletion on loop extension (A) Micro-C contact maps in *PDS5-AID* (SN80) treated with vehicle (+EtOH) or IAA, Δ*wpl1* (KT127), and Δ*wpl1* Δ*eco1* (SN54) cells arrested at metaphase. Vehicle-treated *PDS5-AID* serves as a control. *PDS5-AID* cells were cultures as in Figure S1B. Contact maps were computed on 500 bp-resolution data. The number of valid pairs in each sample was normalized to 42 million. (B) Average contact frequency (represented as observed/expected ratio) between a centromere and a cohesin site on the same chromosome in the indicated strains arrested at metaphase. The centromere-cohesin site pairs were grouped by the distance between the two sites. (C) Contact-versus-distance decaying curves of the contact matrix in the indicated strains, and their first derivatives (slope). (D) Comparison of the centromere-originated loops identified in the indicated samples. (Left) The number of the loops. (Middle) Length distribution. (Right) Scc2 ChIP-seq nFE at the non-centromeric anchor sites. Outliers were not depicted in the boxplots.

Previous studies have reported that the pericentromeric loops are also extended in Δ*wpl1* single deletion mutant and cells in which Pds5 is acutely depleted by auxin-inducible degron system (*PDS5-AID*).^38^^,^^39^^,^^43^ We conducted Micro-C analysis in Δ*wpl1* and Pds5-depleted cells in parallel with Δ*wpl1* Δ*eco1* to compare the characteristics of DNA loops. All the strains were arrested in metaphase. Vehicle-treated *PDS5-AID* strain, as a control, exhibited short DNA loops along the chromosomes, like WT cells (Figures 5A and 5B). Δ*wpl1* and 3-indoleacetic acid (IAA)-treated *PDS5-AID* cells demonstrated the extension of the centromere-originated loops as reported previously but to a lesser extent than Δ*wpl1* Δ*eco1* double deletion mutant (Figures 5A and 5B). The number and length of centromere-originated loops were also moderately increased in Δ*wpl1* and IAA-treated *PDS5-AID* but more prominently in Δ*wpl1* Δ*eco1* (Figure 5D, left and middle). To quantify the extension of centromere-originated loops genome-wide, we grouped the cohesin binding sites based on the distance from the centromere and depicted an aggregated plot for the interaction between the cohesin sites in each group and the corresponding centromere. The interaction became weaker as the cohesin sites were further away from the centromere (Figure 5B). The extent of the interaction in Δ*wpl1* and Pds5-depleted cells reached farther than in WT cells but remained much shorter than in Δ*wpl1* Δ*eco1* double deletion mutant (Figure 5B). The contact-versus-distance decaying curve and its derivative also confirmed a relative increase in long-range contacts of Δ*wpl1* and IAA-treated *PDS5-AID* but not as much as of Δ*wpl1* Δ*eco1* (Figure 5C). Different from Δ*wpl1* Δ*eco1*, Scc2 co-localization was not detected at the anchor cohesin sites in Δ*wpl1* or IAA-treated *PDS5-AID* cells (Figure 5D, right).

Previous studies have shown that the increased long-range interaction in Pds5-depleted cells is comparable to that in Δ*wpl1* Δ*eco1* cells, which is quantitatively different from our results.^38^^,^^39^ These studies used different culture protocols, which may account for the observed differences. Although we confirmed that Pds5 was reduced to less than 10% upon the IAA addition (Figure S1B), it remains possible that a more complete removal of Pds5 could lead to the formation of enlarged pericentromeric DNA loops, similar to those observed in Δ*wpl1* Δ*eco1*.

### Centromere-originated loops in Δ*wpl1* Δ*eco1* are dependent on Scc2

**Figure 6:**
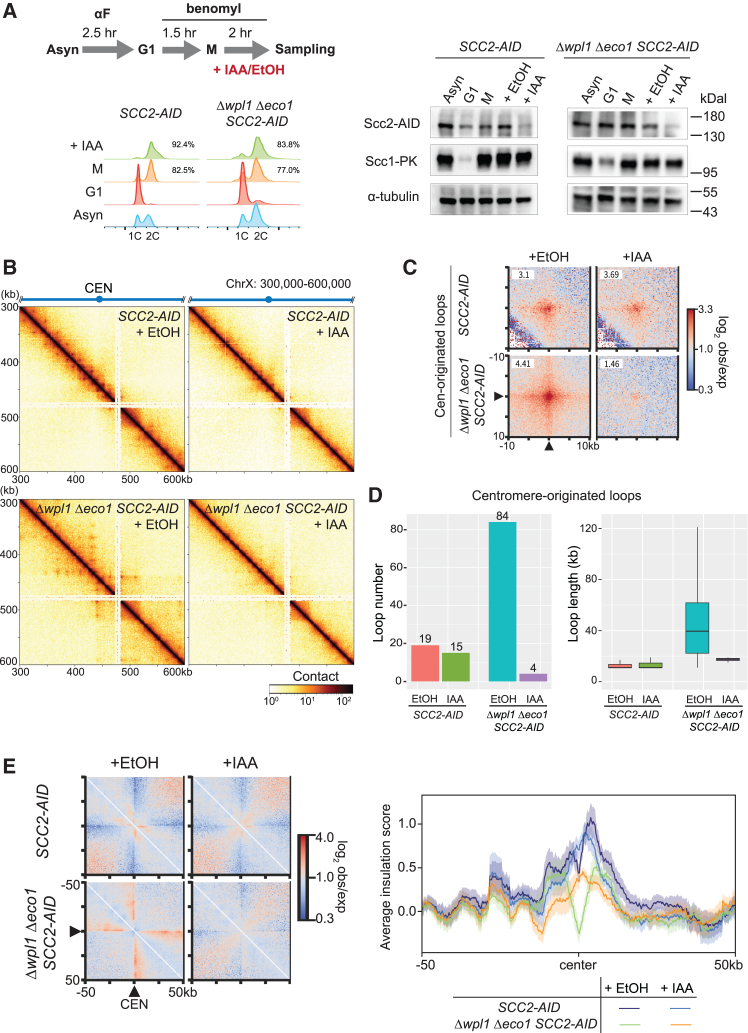
Acute depletion of Scc2 eliminates cohesin-mediated extended loops in Δ*wpl1* Δ*eco1* (A) (Left) Schematic representation of the experimental protocol used to arrest cells in metaphase and induce rapid degradation of Scc2-AID, and cell cycle monitoring by flow cytometry (numbers indicate the proportion of cells with 2C DNA content). (Right) Western blot to verify Scc2-AID depletion. (B) Micro-C contact maps in *SCC2-AID* (SN75) and Δ*wpl1* Δ*eco1 SCC2-AID* (SN74) strains treated with vehicle (EtOH) or IAA at the resolution of 1 kb. The number of valid reads in each sample was normalized to 28 million. (C) Average contact frequency for the centromere-originated loops detected in vehicle-treated *SCC2-AID* and Δ*wpl1* Δ*eco1 SCC2-AID* strains (+EtOH). The averaged contact frequency for the same locus-pairs in IAA-treated condition (+IAA) was shown side by side for comparison. (D) The number and length of centromere-originated loops detected in the indicated conditions. Outliers were omitted from the boxplot. (E) (Left) Average contact frequency around the centromeres. Black triangles indicate the centromere position. (Right) Averaged insulation score around the centromeres in the indicated condition. Bold line, mean; shaded area, 95% confidence interval. See also Figures S16 and S17.

The co-localization of Scc2 and cohesin in Δ*wpl1* Δ*eco1* implied that the extended pericentromeric DNA loops were generated by the Scc2-dependent loop extrusion activity of cohesin. To verify this, we investigated chromosome folding in cells where Scc2 was acutely depleted by the AID system. First, we verified the efficacy of AID-induced Scc2 depletion. In budding yeast, cohesin is almost absent in α-factor-arrested G1 cells and loaded onto chromosomes during late G1–S phase. When G1-arrested cells were released and re-arrested at metaphase in the presence of IAA, we observed very little cohesin loading at all the cohesin sites in the genome (Figure S16A, +IAA(S)). This clearly indicates that the AID system successfully depleted Scc2, which is required for cohesin loading onto chromosomes, to a very low level. We then tested another condition, where Scc2 was depleted after metaphase arrest (Figures 6A and S16A, +IAA). In this condition, cohesin loading onto the chromosome arms was only marginally attenuated, indicating that the latter experimental scheme allows the suppression of Scc2 activity in metaphase without affecting sister chromatid cohesion formation in S phase. Scc1 binding level at the centromeres was greatly reduced under the second condition, particularly in Δ*wpl1* Δ*eco1* (Figure S16A, right). This is consistent with the fact that centromeres are active cohesin loading sites in metaphase cells lacking spindle tension.^50^

We conducted Micro-C analysis of the cells where Scc2 was depleted after metaphase arrest. As a control, we analyzed Δ*wpl1* Δ*eco1 SCC2-AID* strain (strain SN74) in the absence of IAA, which revealed some changes in the contact map compared with the untagged Δ*wpl1* Δ*eco1* strain (KT110) (Figure S17A). Of particular, loops connecting the cohesin sites on chromosome arms were detected in addition to the centromere-originated extended loops, and the number of detected loops was larger in SN74 (Figure S17B, left). These differences were reproducibly observed, implying that the difference was not due to experimental variability. Nevertheless, the overall trend in chromosome folding change was identical between the two strains; both KT110 and SN74 exhibited significantly longer loops than WT (Figure S17B, right) and a similar decrease in short-range contacts accompanied by an increase in long-range contacts (Figure S17C). In addition, average contact maps around centromeres in SN74 portrayed the loss of centromere-neighboring domains and the emergence of long-range centromere-originated interactions, like KT110 (Figure S17D). We hence concluded that untagged Δ*wpl1* Δ*eco1* and Δ*wpl1* Δ*eco1 SCC2-AID* strains shared the same characteristic in centromere-involved chromosome folding and used SN74 strain for subsequent experiments.

The Micro-C contact map revealed that depletion of Scc2 in metaphase-arrested Δ*wpl1* Δ*eco1* cells resulted in the disappearance of the centromere-originated loops (Figure 6B); they were greatly reduced in contact frequency, number, and length (Figures 6C and 6D). In WT, the same Scc2 depletion showed only a modest impact on the centromere-originated loops (Figures 6C and 6E), suggesting that a lower level of functional Scc2 is sufficient for loop formation in WT cells. Scc2 depletion also affected the additional arm-to-arm loops found in SN74 strain, because off-diagonal APA plots for all cohesin-bound loop anchors showed that the average genome-wide contact frequency was drastically reduced in Δ*wpl1* Δ*eco1* (from 3.14 to 1.61) (Figure S16C). On-diagonal APA analysis and average insulation score profile around centromeres indicate that Scc2 depletion in metaphase resulted in the loss of the insulating feature of centromeres in Δ*wpl1* Δ*eco1* (Figure 6E). Taken together, we conclude that the Scc2 activity in metaphase is required for the formation of cohesin-mediated loops and the insulation across the centromeres observed in Δ*wpl1* Δ*eco1.*

### Extended pericentromeric loops impede chromosome segregation process

**Figure 7:**
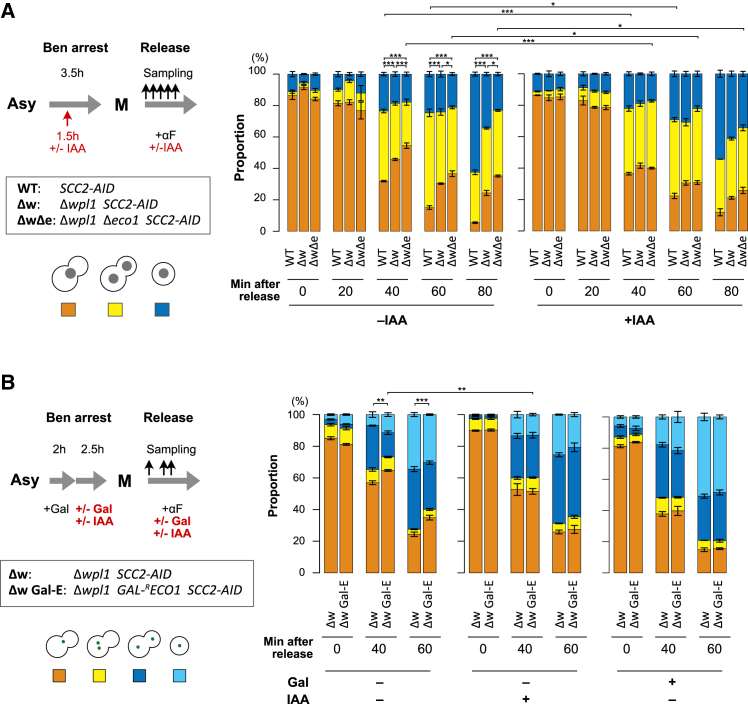
Expanded pericentromeric loops impeded the smooth progression of chromosome segregation (A) (Left) Schematic representation of the experimental protocol to make cells proceed synchronously through mitosis in the absence or presence of Scc2. *SCC2-AID* (SN75), Δ*wpl1 SCC2-AID* (ST726), and Δ*wpl1* Δ*eco1 SCC2-AID* (SN74) were analyzed. Ben, benomyl; αF, α factor. (Right) Proportion of budded cells with one nucleus, budded cells with two nuclei, and unbudded cells with one nucleus are depicted. The mean of three or more biological replicates, in each of which >150 cells were counted, are shown. Error bar, SEM. Two-tailed t test was conducted to evaluate the statistical significance of the difference in the fraction of budded cells with one nucleus (orange). ∗∗∗, *p* < 0.001; ∗, *p* < 0.05. (B) (Upper left) Schematic representation of the experimental protocol to make cells proceed synchronously through mitosis in the absence or presence of Eco1. Δ*wpl1 SCC2-AID* (ST730) and Δ*wpl1 GAL-*^*R*^*ECO1 SCC2-AID* (ST731) were analyzed. Gal, galactose. (Lower left) Categorization of cells based on morphology and *URA3*-GFP foci. (Right) The proportion of cells in each category. Cells were collected at 0, 40, or 60 min after the release from benomyl-arrest. The means of three biological replicates, in each of which >150 cells were counted, are shown. Error bar, SEM. Two-tailed t test was conducted to evaluate the statistical significance of the difference in the fraction of budded cells with a single GFP dot (orange). ∗∗∗, *p* < 0.001; ∗∗, *p* < 0.01.

We explored how the extension of the pericentromeric DNA loops affects the function of the kinetochore. WT and Δ*wpl1* Δ*eco1* strains possessing *SCC2-AID* were arrested in metaphase with benomyl and released from the arrest, followed by DAPI-based monitoring of nuclear division (the detailed experimental scheme is in Figure 7A, left). In WT, anaphase or telophase cells, or cells with segregated nuclei, started to appear 40 min after the release (Figure 7A, right). In contrast, Δ*wpl1* Δ*eco1* cells exhibited a delay in nuclear division because the proportion of the anaphase or telophase cells was significantly lower than that of WT 40 min after the release or later. Consistently, the proportion of large-budded cells with a single nucleus remained high, compared with that in WT. Δ*wpl1* mutant showed a milder delay in nuclear division than Δ*wpl1* Δ*eco1* double mutant, which is consistent with the notion that Wpl1 and Eco1 additively restrict the size of the pericentromeric loops. Of note, the delay in nuclear division was rescued to some extent by metaphase-specific depletion of Scc2. This result suggests that the extended DNA loops around centromeres, of which the formation is dependent on Scc2’s function in metaphase, impede the smooth progression of nuclear division. On the other hand, we observed that Scc2 depletion caused a delay in nuclear division in WT cells. This implies that cohesin-mediated chromosome folding in WT may contribute positively to chromosome segregation.

We conducted another experiment to further verify the physiological relevance of the pericentromeric loop size restriction. Δ*wpl1 GAL-*^*R*^*ECO1* strain was arrested in metaphase with benomyl, and the medium was changed to galactose-free YPD to repress Eco1 expression. Then, the cells were released from the arrest, and chromosome segregation was monitored by the TetO/TetR-GFP mark at *URA3* locus (experimental scheme in Figure 7B, left). At metaphase, Δ*wpl1* Δ*eco1* shows a higher proportion of cohesion-defective cells than Δ*wpl1*.^26^ However, we observed little difference in the proportion of cohesion-defective cells between Δ*wpl1 GAL-*^*R*^*ECO1* and the control Δ*wpl1* at the timing of release (0 min). This indicates that Δ*wpl1 GAL-*^*R*^*ECO1* cells passed through S phase with functional Eco1, and sister chromatid cohesion was established with little defect. At 40 and 60 min after the release, Δ*wpl1 GAL-*^*R*^*ECO1* showed a significantly higher proportion of the cells with unsegregated GFP focus than Δ*wpl1*, indicating a delay in mitotic progression (Figure 7B, right). The delay was not observed when Scc2 was degraded in metaphase or when Eco1 was continuously expressed in a galactose-containing medium. Since the degree of the pericentromeric DNA loop extension was more prominent in Δ*wpl1* Δ*eco1* than Δ*wpl1*, this data also supports the notion that the extended pericentromeric loops are detrimental to timely chromosome segregation. In this experiment, we observed no decrease in chromosome segregation fidelity, which would be indicated by an increase in the frequency of G1 phase cells with two GFP dots. To clarify how the enlarged pericentromeric loop affects the fidelity of chromosome segregation, further investigation using a more sophisticated experimental system would be necessary.

## Discussion

In WT budding yeast, Scc2 shows no co-localization with cohesin on chromosome arms.^45^^,^^47^ We found that Scc2 co-localized with cohesin at cohesin binding sites in Δ*wpl1* Δ*eco1* strain, suggesting that Scc2 associates with cohesin more stably in this condition. Structural and biochemical studies suggest that cohesin acetylated by Eco1 loses its affinity for Scc2.^32^^,^^33^ Our quantitative analysis of the ChIP-seq data (Figure 2) revealed that Eco1 deletion indeed enhanced the Scc2-cohesin interaction on chromosomes, but only marginally in cells lacking Eco1 alone; simultaneous deletion of Eco1 and Wpl1 resulted in a more substantial increase of the Scc2-cohesin interaction. We also found that the Scc2-cohesin co-localization occurred only in benomyl-treated metaphase cells, and mitosis-specific depletion of Eco1 was sufficient to promote the co-localization and the concomitant decrease of Smc3 acetylation level in Δ*wpl1* cells. These results strongly suggest that Eco1 acetylates Smc3 to weaken the Scc2-cohesin interaction in metaphase cells without spindle tension.

Wpl1 is known to promote the dissociation of non-acetylated cohesin from chromosomes.^52^ In fact, the height of the cohesin peak was reduced by 70% in the cells lacking Eco1 alone, compared to WT. This reduction partially explains why Scc2 co-localization with cohesin was not detected in the singly Eco1-depleted cells using ChIP-seq. Eco1 depletion alone, however, did not increase the affinity of Scc2 for cohesin to a level comparable to that observed in Δ*wpl1* Δ*eco1* (Figure 2B), suggesting that Wpl1 has an additional, more direct role in the control of Scc2-cohesin interaction. Recent structural predictions by AlphaFold2 imply the possibility that Wpl1 interacts with both Pds5 and cohesin to form ternary complexes.^53^ Δ*wpl1* single deletion mutant actually caused a substantial reduction of Pds5 binding at cohesin sites (Figure S5C). Thus, Wpl1 deletion is likely to weaken the Pds5-cohesin interaction, thereby promoting Scc2 binding to cohesin.

In mitotically arrested WT cells lacking the spindle, centromere-anchored DNA loops of ∼10 kb in length are observed.^44^ These loops are thought to be formed by cohesin bound to the centromeres and catalyzing the DNA loop extrusion reaction. We observed centromere-anchored DNA loops of more than 100 kb in length in Δ*wpl1* Δ*eco1* strain. The presence of Scc2 has been shown to promote loop extrusion activity *in vitro.*^8^ Based on this observation, we propose that the extended pericentromeric loops observed in the Δ*wpl1* Δ*eco1* strain are a consequence of enhanced loop extrusion activity. Scc2-associated cohesin remains ATPase active and continuously exhibits the loop extrusion reaction, resulting in expanded DNA loops. Strong genes transcribed toward the centromere could act as a barrier to loop expansion, but cohesin that maintains activity is assumed to overcome the barriers one by one, allowing continuous loop expansion. This idea is supported by the observations that the extended pericentromeric loops were not detected after the removal of Scc2 in mitotic cells and that the amount of Scc2 co-localized with cohesin is inversely correlated with the distance from the centromere. Our result is consistent with the previous finding that expanded DNA loops in Pds5-depleted cells with *scc3-K404E* mutation are also dependent on Scc2.^43^ Thus, we conclude that Eco1 and Wpl1 additively contribute to the size restriction of pericentromeric DNA loops.

Recently, an alternative model, wherein cohesin forms loop structures on chromosomes independently of loop extrusion (referred to as the loop capture model), has been proposed.^8^ While our findings are consistent with the loop extrusion mechanism, they do not exclude the possibility that the extended pericentromeric loops are generated via the loop capture mechanism. In this scenario, Scc2 binding could facilitate cohesin’s loop capture activity, thereby enabling the formation of more extended loops. Further experiments are necessary to distinguish between these two models.

In WT cells, re-orientation of the loop boundary genes impeded the establishment of chromosome bipolar attachment.^44^ In this study, we demonstrated that Δ*wpl1* Δ*eco1* double deletion mutant exhibited a delay in mitotic chromosome segregation, revealing that *trans-*acting factors also play a role in facilitating the smooth progression of chromosome segregation through the restriction of the pericentromeric DNA loop size. As suggested by Paldi et al.,^44^ the expanded pericentromeric DNA loops may disrupt sister chromatid cohesion in wider chromosome regions, thereby disturbing chromosome segregation. In addition, the presence of large DNA loops near the centromeres may directly hinder kinetochore-microtubule attachment. Δ*wpl1* Δ*eco1* mutant is highly sensitive to a microtubule depolymerization drug, benomyl.^26^ Under conditions that interfere with centromere-spindle interaction, regulation of the pericentromeric loop size may be particularly important.

Eco1 is essential for the establishment of sister chromatid cohesion during S phase.^54^ In this study, we observed that mitosis-specific depletion of Eco1 in Δ*wpl1* strain was sufficient to cause the Scc2 co-localization with cohesin and a delay in chromosome segregation similar to that seen in Δ*wpl1* Δ*eco1* strain. This implies that Eco1 must function in metaphase to restrict pericentromeric DNA loop expansion. Consistently, Eco1 reportedly remains active in cohesin acetylation even after DNA replication is completed.^24^

A very recent report revealed that purified yeast cohesin with Scc2-Scc4 exhibits DNA loop extrusion activity almost equivalent to that of human cohesin *in vitro*.^8^ Our current study demonstrated that yeast cohesin is actually capable of forming large DNA loops of several hundred kb *in vivo*, comparable to those in human nuclei, if Scc2-cohesin interaction is free from inhibitory regulation by Wpl1 and Eco1. Wpl1 and Eco1 additively suppress long-range interactions also in meiotic cells.^55^ We suppose that meiotic cell utilizes a similar regulation mechanism of the Scc2-cohesin interaction by Wpl1 and Eco1. It is notable that co-localization of Scc2 and cohesin was not observed in Δ*wpl1* Δ*eco1* cells during the S phase or metaphase under spindle tension. Other factors may regulate cohesin specifically at unattached centromeres in metaphase cells so that it exhibits higher affinity to Scc2 and becomes competent to DNA loop extrusion. It will be interesting to explore further how the Scc2-cohesin interaction is regulated in a cell cycle- and context-dependent manner.

### Limitations of this study

This study provides insights into how Wpl1 and Eco1 regulate the Scc2-cohesin interaction to restrict pericentromeric loop size. However, our observation is solely based on calibrated ChIP-seq analysis; we measured Scc2 binding intensity at cohesin binding sites in the genome and inferred the strength of the Scc2-cohesin interaction from these data. Future studies should verify our findings using biochemical approaches that can more directly detect the Scc2-cohesin interaction. Additionally, the molecular mechanism by which Wpl1 and Eco1 prevent the Scc2-cohesin interaction remains to be elucidated, which could be addressed through biochemical and structural studies.

## Resource availability

### Lead contact

Further information on resources and requests should be directed to and will be fulfilled by the lead contact, Takashi Sutani (tsutani@iqb.u-tokyo.ac.jp).

### Material availability

All materials generated in this study are available from the lead contact without restriction.

### Data and code availability

• All the sequencing data for ChIP-seq and Micro-C are available through GEO: GSE248144. Original data of western blotting, flow cytometry, and qPCR have been deposited to Mendeley Data: https://data.mendeley.com/datasets/9gy6p7whwv/1.
• This article does not report original code. The scripts used for the sequencing data analysis are available from the lead contact upon request.
• Any additional information required to reanalyze the data reported in this article is available from the lead contact upon request.

## Acknowledgments

We thank Dr. Kristian Jeppsson and all members of Shirahige laboratory for discussion. This work was supported by JST CREST grant number JPMJCR18S5, 10.13039/501100001691JSPS KAKENHI grant numbers JP20H05940, JP20H05933 and JP20H05686, AMED BINDS grant number 22ama121020j0001, AMED ASPIRE-A grant number JP23jf0126003, and Swedish Research Council registration number 2022-03478 (to K.S.); 10.13039/501100001691JSPS KAKENHI grant number JP21K06012 (to T.Su.).

## Author contributions

S.A.N., K.S., and T. Sutani designed the study and interpreted the data; S.A.N. and T. Sutani performed the experiments; S.A.N. and T. Sakata analyzed data; S.A.N. and T. Sutani wrote the manuscript; S.A.N., T. Sakata, K.S., and T. Sutani reviewed and edited the manuscript.

## Declaration of interests

The authors declare no competing interests.

## Declaration of generative AI and AI-assisted technologies in the writing process

During the preparation of this work, the authors used DeepL Pro and Grammarly in order to improve readability and language. After using these tools, the authors reviewed and edited the content as necessary and took full responsibility for the content of the publication.

## STAR★Methods

### Key resources table

**Table:** 

REAGENT or RESOURCE	SOURCE	IDENTIFIER
**Antibodies**		

Anti V5-Tag Antibody, clone SV5-Pk1	Bio-Rad	Cat# MCA1360; RRID: AB_322378
Monoclonal anti-AID/IAA17	Cosmo Bio	CAC-APC004AM-T
Monoclonal anti-Rpo21, 8WG16 clone	Sigma-Aldrich	05-952-I
Anti-acetylated Smc3	Beckouët et al., 2010^56^	N/A

**Chemicals, peptides, and recombinant proteins**		

α-factor	Synthesized by Eurofins Genomics	N/A
PRONASE Protease, Streptomyces griseus	Calbiochem	9036-06-0
Benomyl	Sigma Aldrich	17804-35-2
Indole-3-acetic acid (IAA)	Sigma Aldrich	I2886
Zymolyase 100T	Nacalai Tesque	07665-55
Micrococcal Nuclease (MNase)	NEB	M0247S
Pierce DSG, No-Weigh Format	Thermo Scientific	A35392
NEBuffer 1	New England Biolabs	B7001S
NEBuffer 2	New England Biolabs	B7002S
ATP solution (100mM)	Thermo Scientific	R0441
BSA, Molecular Biology Grade	New England Biolabs	B9000S
T4 Polynucleotide Kinase	New England Biolabs	M0201L
DNA polymerase Large Klenow Fragment	New England Biolabs	M0210L
10 mM dTTP	New England Biolabs	N0443S
10 mM dGTP	New England Biolabs	N0442S
1 mM biotin-14-dATP	Jena Bioscience	NU-835-BIO14-L
1 mM biotin-14-dCTP	Jena Bioscience	NU-956-BIO14-L
T4 DNA ligase buffer	New England Biolabs	B0202S
T4 DNA ligase	New England Biolabs	M0202L
NEB Exonuclease III	New England Biolabs	M0206S
AMPure XP beads	Beckman Coulter	A63881
Dynabeads™ MyOne™ Streptavidin C1	Invitrogen	65001
Dynabeads™ Protein A	Invitrogen	10002D

**Critical commercial assays**		

KAPA SYBR Fast qPCR kit	Sigma Aldrich	KR0389
ZymoClean and Concentration Kit	ZymoResearch	D4014
ZymoClean Gel DNA Recovery Kit	ZymoResearch	D4007
NEBNext Ultra II DNA library Prep Kit for Illumina	NEB	E7645
QIAquick PCR purification kit	QIAGEN	28104

**Deposited data**		

ChIP-seq and Micro-C, raw and processed data	NCBI GEO	GSE248144

**Experimental models: Organisms/strains**		

*S*. *cerevisiae*: BY4741 *MATa his3Δ1 leu2Δ0 met15Δ0 ura3Δ0 trp1Δ*	Lab stock	SKY001
*S*. *cerevisiae*: SKY001 *wpl1Δ::LEU2*	Sutani et al., 2009^26^	KT127
*S*. *cerevisiae*: SKY001 *wpl1Δ::LEU2 eco1Δ::TRP1*	Sutani et al., 2009^26^	KT110
*S*. *cerevisiae*: SKY001 *SCC2-9PK:TRP1*	This study	SN40
*S*. *cerevisiae*: SKY001 *wpl1Δ::LEU2 eco1Δ::TRP1 SCC2-9PK:HIS3MX6*	This study	SN54
*S*. *cerevisiae*: SKY001 *wpl1Δ::LEU2 SCC2-9PK:HIS3MX6*	This study	SN53
*S*. *cerevisiae*: SKY001 *SCC2-9PK:TRP1 pGAL1-3HA-CDC20:hphMX6*	This study	TS732
*S*. *cerevisiae*: SKY001 *wpl1Δ::LEU2 eco1Δ::TRP1 SCC2-9PK:HIS3MX6 pGAL1-3HA-CDC20:hphMX6*	This study	TS733
*S*. *cerevisiae*: SKY001 *SCC2-9PK:TRP1 pGAL-Ub-*^*R*^*ECO1:kanMX6*	This study	SN41
*S*. *cerevisiae*: SKY001 *wpl1Δ::LEU2 SCC2-9PK:TRP1 pGAL-Ub-*^*R*^*ECO1:kanMX6*	This study	SN722
*S*. *cerevisiae*: SKY001 *SCC1-9PK:kanMX6*	This study	SN27
*S*. *cerevisiae*: SKY001 *SCC1-9PK:TRP1 pGAL-Ub-*^*R*^*ECO1:kanMX6*	This study	SN39
*S*. *cerevisiae*: SKY001 *SCC2-9PK:TRP1 AUR1:TIR1-9Myc,AUR1-C PDS5-AID:kanMX6*	This study	SN80
*S*. *cerevisiae*: SKY001 *PDS5-9PK:HIS3MX6*	This study	SN47
*S*. *cerevisiae*: SKY001 *wpl1Δ::LEU2 eco1Δ::TRP1 PDS5-9PK:HIS3MX6*	This study	SN48
*S*. *cerevisiae*: SKY001 *wpl1Δ::LEU2 PDS5-9PK:HIS3MX6*	This study	SN49
*S*. *cerevisiae*: SKY001 *wpl1Δ::LEU2 eco1Δ::TRP1 AUR1:TIR1-9Myc,AUR1-C SCC2-AID:kanMX6 SCC1-9PK:HIS3MX6*	This study	SN74
*S*. *cerevisiae*: SKY001 *AUR1:TIR1-9Myc,AUR1-C SCC2-AID:kanMX6 SCC1-9PK:HIS3MX6*	This study	SN75
*S*. *cerevisiae*: SKY001 *wpl1Δ::LEU2 AUR1:TIR1-9Myc,AUR1-C SCC2-AID:kanMX6 SCC1-9PK:HIS3MX*	This study	ST726
*S*. *cerevisiae*: W303-1A *ura3::URA3,tetOs his3::HIS3,tetR-GFP wpl1Δ::LEU2 AUR1:TIR1-9Myc,AUR1-C SCC2-AID:hphMX6*	This study	ST730
*S*. *cerevisiae*: W303-1A *ura3::URA3,tetOs his3::HIS3,tetR-GFP wpl1Δ::LEU2 pGAL-Ub-*^*R*^*ECO1:kanMX6 AUR1:TIR1-9Myc,AUR1-C SCC2-AID:hphMX6*	This study	ST731
*Candida glabrata*, *SCC1-9PK-natMX*	Hu et al., 2015^57^	K23308

**Oligonucleotides**		

Primers for quantitative PCR. See Table S1.	N/A	N/A

**Software and algorithms**		

Bowtie	Langmead et al., 2009^58^	https://bowtie-bio.sourceforge.net/index.shtml
DROMPA	Nakato et al., 2013^59^	https://drompaplus.readthedocs.io/en/latest/index.html
deepTools	Ramírez et al., 2016^60^	https://deeptools.readthedocs.io/en/latest/
Integrative Genomics Viewer	Robinson et al., 2011^61^	https://igv.org
Juicer Tools	Durand et al., 2016^62^	https://www.encodeproject.org/software/juicertools/
Galaxy HiCExplorer	Wolff et al., 2020^63^	https://hicexplorer.usegalaxy.eu
Coolpup.py	Flyamer et al., 2020^64^	https://coolpuppy.readthedocs.io/en/latest/walkthrough.html
Cooler	Abdennur & Mirny, 2020^65^	https://github.com/open2c/cooler?tab=readme-ov-file#Installation
pairtools	Open 2C et al., 2024^66^	https://pairtools.readthedocs.io/en/latest/
cooltools	Open 2C et al., 2024^67^	https://cooltools.readthedocs.io/en/latest/
R	R Core Team, 2021^68^	https://www.r-project.org

### Experimental model and study participant details

#### Yeast strains

*S. cerevisiae* strains used in this study are wild-type BY4741 and its derivatives, except the strains to visualize *URA3* locus by TetO/TetR-GFP mark, which are W303-1A derivatives. They are listed in the key resources table. Epitope tagging, *aid* module tagging, and gene deletion were performed by one-step PCR-based strategy.^69^^,^^70^ Correct tagging or deletion of the target gene was checked by PCR. The tagging was also confirmed by western blotting.

### Method details

#### Cell culture

Yeast cells were cultured in YPD medium^71^ at 23°C unless otherwise mentioned. To be arrested in metaphase, cells were cultured in medium containing 80 μg/mL benomyl (Sigma-Aldrich) for 2.5 h. For synchronization in S phase, cells were first arrested in G1 phase by culturing in medium containing 2 μM α-factor (peptide synthesized by Eurofins Genomics) for 2.5 h. Then, cells were released from the arrest by transferring them to α-factor-free medium containing 150 μg/mL of Pronase (Calbiochem) and cultured for 30 min. To synchronize cells in metaphase by Cdc20 depletion, *GAL-CDC20* cells were cultured in YPRG (YPD lacking glucose, supplemented with 2% raffinose and 0.1% galactose) to reach mid-log phase, transferred to YPD medium, and incubated at 23°C for 2.5 h for Cdc20 depletion. To prepare metaphase-arrested cells lacking Scc2 protein, which were used for Micro-C analysis, *SCC2-AID* cells arrested in G1 phase by α-factor were released into the medium containing 150 μg/ml Pronase, 80 μg/mL benomyl and cultured for 1.5 h. Then, 3-indoleacetic acid (IAA) (Sigma-Aldrich, I2886) was added to the final concentration of 0.2 mg/mL and cultured for an additional 2.5 h. For the stock solution, IAA was dissolved in ethanol at 20 mg/ml. For vehicle-treated control cells, ethanol was added to the final concentration of 1% (v/v) instead of IAA. To make cells proceed through S phase without Scc2 and arrest at metaphase, *SCC2-AID* cells arrested in G1 phase by α-factor were released into the medium containing 150 μg/ml Pronase, 80 μg/mL benomyl, and 0.2 mg/mL IAA and cultured for 2.5 h. To monitor chromosome segregation in the absence of Scc2, asynchronous *SCC2-AID* cells were cultured in the medium containing 80 μg/mL benomyl for 1.5 h, followed by the addition of 0.2 mg/mL IAA and cultivation for an additional 2 h. Then, the cells were released from the arrest by transferring into benomyl-free medium containing 0.2 mg/mL IAA and 2 μM α-factor. To deplete Pds5 protein in metaphase-arrested cells, *PDS5-AID* cells arrested in G1 phase by α-factor were released into the medium containing 150 μg/ml of Pronase, 80 μg/mL benomyl, and 0.2 mg/mL IAA and cultured for 2.5 h. To prepare the cells that proceeded through S phase without Eco1 protein and arrested in metaphase (used for ChIP-seq analysis), we utilized *GAL-*^*R*^*ECO1* strain,^48^ where Eco1 with short protein half-life is expressed from the galactose-dependent *GAL1* promoter. The cells grown in YPRG medium at 30°C were transferred to YPD and cultured for 1 h at 23°C, followed by addition of 2 μM α-factor and cultivation for additional 2.5 h. Then, the cells were released into α-factor-free medium containing 150 μg/ml Pronase, and 80 μg/mL benomyl and cultured for 2.5 h. To prepare metaphase-arrested cells that lacked Eco1 after DNA replication completion (used for ChIP-qPCR and chromosome segregation analysis), asynchronous *GAL-*^*R*^*ECO1* cells were cultured in YPRG containing 80 μg/mL benomyl at 30°C for 2 h, then transferred into YPD medium containing 80 μg/mL benomyl and cultured at 23°C for 2.5 h. To induce Eco1 in metaphase-arrested cells, *Δwpl1 GAL-*^*R*^*ECO1* cells were cultured to mid-log phase in YPR (YPD lacking glucose and supplemented with 2% raffinose) and arrested in metaphase by adding benomyl to a final concentration of 80 μg/mL. Subsequently, galactose was added to the culture at a final concentration of 2% to induce the expression of Eco1 for 1 hour. To observe chromosome segregation phenotype, the metaphase-arrested cells were released into benomyl-free YPD medium containing 2 μM α-factor at 23°C. The control cells that continue to express Eco1 were prepared by using YPRG medium continuously instead of YPD at 30°C.

#### Flow cytometry

Cell cycle synchronization was monitored by propidium iodide flow cytometry analysis^70^ with the following details. Cells were fixed in 75% ethanol at 4°C overnight before incubating at 50°C with 0.25 mg/mL RNAase (Sigma-Aldrich, 10109142001) for 1 h and 0.25 mg/mL Proteinase K (GOLDBIO, P-480-100) for 1 h. Then, cells were pelleted and resuspended into sodium citrate pH 7.4 containing 8 μg/mL propidium iodide and incubated for 30 min at room temperature for DNA staining. The analysis was conducted on a FACSCalibur or Accuri C6 flow cytometer (BD Biosciences).

#### Protein analysis

Gene tagging and protein degradation were monitored by western blotting. Yeast lysate was prepared using the trichloroacetic acid (TCA) method.^70^^,^^72^ ∼5×10^7^ yeast cells were washed by distilled water, resuspended with 50 μL of 20% TCA solution, and broken with glass beads using multi-beads shocker (Yasui-kikai). The cell lysate was transferred to a 1.5 mL-tube, and the protein was pelleted by centrifugation. The pellet was resuspended with 100 μL of 2× Laemmli buffer (0.125 M Tris-HCl (pH 6.8), 4% SDS, 20% glycerol, 10% 2-mercaptoethanol, 0.004% bromophenol blue) and neutralized by adding 50 μL of 1 M Tris-HCl (pH 8.0). The samples were then boiled at 95°C for 5 min and subjected to SDS-PAGE. SDS-PAGE, and Western blotting were performed following the standard protocol.^73^ Western blot image was acquired by ImageQuant LAS 4000 (GE Healthcare) or Fusion FX (Vilber). Antibodies used for ChIP and Western blotting are described in the KEY RESOURCES TABLE.

#### Cell imaging

For fixation, 1 mL of cell culture was fixed with 3.7% formaldehyde (Fujifilm, 064-00406) for 20 min at room temperature. Then, the cells were pelleted, resuspended in 1 mL of 100% ethanol (Fujifilm, 057-00456), and incubated at 4°C for 1 h for additional fixation. The cells were washed twice with TBS, followed by nuclear staining in 50 μL of PBS containing 2 μg/mL DAPI (Dojindo, 340-07971) for 5 min. Finally, the cells were resuspended in 50 μL of PBS and observed by Olympus IX-83 fluorescence microscope equipped with DP23M digital camera.

#### Chromatin immunoprecipitation (ChIP)

ChIP was performed as described previously^74^ with the following details. 100 mL of cell culture were cross-linked in 1% formaldehyde for 30 min at room temperature, then incubated at 4°C overnight. For chromatin extraction, cell pellets were resuspended into lysis buffer (50 mM Hepes-KOH pH 7.5, 140 mM NaCl, 1 mM EDTA, 1% Triton-X100, 0.1% Na-deoxycholate, immediately before use add 1X protease inhibitor complete cocktail (Roche, 11836145001) and 1 mM PMSF), cells were broken using multi-beads shocker (Yasui-kikai), and all cell extract was collected by centrifugation. Cell extract was then processed to chromatin shearing by sonication (Sonifier Branson, 2508). Fragmented chromatin was incubated overnight at 4°C with antibody-conjugated Dynabeads Protein A (Invitrogen, 10002D). After completing immunoprecipitation, beads were washed, and chromatin was eluted and processed to crosslink reverse. DNA was clean-up by adding 50 μL of TE buffer containing 10 μg RNaseA (Sigma-Aldrich, 10109142001) and incubating for 1 hour at 37°C, followed by adding 10 μg of proteinase K (GOLDBIO, P-480-100) and incubating for 2 h at 37°C. DNA was purified by QIAquick PCR purification kit (Qiagen, 28106) according to standard instructions from the manufacturer. Purified DNA was subjected to quantitative PCR or sequencing library preparation. To conduct calibrated ChIP-seq, we utilized the *Candida glabrata* strain in which the endogenous *SCC1* gene was tagged with PK epitopes (a gift from Prof. Kim Nasmyth). Every 2 (or 5 in the case of Scc2 ChIP-seq) OD units of *S. cerevisiae* cell culture were mixed with 1 OD unit of *C. glabrata* asynchronous cell culture immediately before fixation.

#### ChIP-seq library preparation and sequencing

Both input and ChIP fractions of DNA were processed. Purified DNA was further fragmented to the size of ∼150 bp by the Covaris focused-ultrasonicator (Covaris M220) and subjected to library preparation using the NEBNext Ultra II DNA Library Prep Kit for Illumina (NEB, E7645). Sequencing of the library was performed using Illumina HiSeq2500 and NextSeq2000 to produce 65-bp single-end reads and 36-bp paired-end reads, respectively.

#### ChIP-qPCR

Analysis of ChIP-purified DNA by quantitative PCR (qPCR) was performed using KAPA SYBR Fast qPCR kit (KAPA Biosystems) and StepOnePlus real-time PCR systems (Life Technology, Inc.) as per manufacturers’ instructions. Input and ChIP DNA were measured in duplicates. The primers used in qPCR are listed in Supplemental Table S1.

#### Micro-C library preparation

Micro-C protocol was based on Costantino et al.^38^ and Hsieh et al.^75^ with minor modifications. Briefly, 100 mL of culture at OD ∼0.8 was fixed with 3% formaldehyde for 15 min at 30°C. Fixation was quenched by adding glycine to a final concentration of 0.2 M and shaking for 5 min at 30°C. Cells were collected and washed with sterile, ice-cold water before processing to cell-wall permeabilization by resuspending to 10 mL buffer Z (1 M sorbitol, 50 mM Tris-HCl pH 7.4, supplemented with 10 mM 2-mercaptoethanol (Wako, 133-14571)) containing 250 μg/mL Zymolyase 100T (Nacalai Tesque, 07665-55), and shaking at 180 rpm for 40 min at 30°C. The spheroplasts were collected and washed with PBS, then transferred to a 1-mL DNA LoBind tube (Eppendorf, 022431021). Cells were resuspended to 1 mL PBS, and 10 μL of 0.3 M DSG (Thermo Fisher Scientific, A35392) was added to the final concentration of 3 mM for the second cross-linking. The tube was shaken for 40 min at 30°C before quenching by 0.2 M glycine for 5 min at 30°C on a shaking incubator. Cells were washed by PBS and pelleted by centrifugation. The cell pellets can be snap-frozen in liquid nitrogen and stored at -80°C.

For chromatin fragmentation by MNase, cell pellets were first incubated for 5 min in 200 μL ice-cold MB1 (50 mM NaCl, 10 mM Tris-HCl pH 7.5, 5 mM MgCl_2_, 1 mM CaCl_2_, 0.075% NP-40 and 1× cOmplete EDTA-free protease inhibitor cocktail (Roche, 04693132001)). ∼1,000 U of MNase (NEB, M0247S) was added to a cell sample derived from 100-mL cell culture, and the mixture was incubated for 20 min at 37°C, which produced ∼90% mono-nucleosome and ∼10% di-nucleosome. The reaction was stopped by adding 5 μL of 0.1 M EGTA (final conc. 2.5 mM), incubated at 65°C for 10 min. In-nucleus fragmented chromatin was pelleted and washed twice by MB2 (50 mM NaCl, 10 mM Tris-HCl pH 7.5, 10 mM MgCl_2_) before processing to end-preparation.

A 3-step end-preparation was conducted according to Costantino et al*.*^38^ The reaction was inactivated by adding EDTA to the final concentration of 30 mM and incubating for 20 min at 65°C. Chromatin was washed once by 1× T4 DNA ligase buffer (NEB, B0202) and pelleted by centrifugation.

For proximity ligation, the pellet was resuspended into 500 μL of ligation mix (1× T4 DNA ligase buffer, 1× BSA (NEB, B9000S), 5000 CEU T4 DNA ligase (NEB, M0202L)) and rotated on a slow rotator for 3 h or more at room temperature. The biotin-dNTP at unligated ends was then removed by incubation with 200 U Exonuclease III (NEB, M0206S) in 1× NEB buffer 1 (NEB, B7001S) for 15 min at 37°C. In the next step, chromatin was processed to reverse crosslinking by incubating at 65°C overnight in TE supplemented with 1% SDS, 15 μg/mL Proteinase K, and 15 μg/mL RNase A. DNA was purified using standard phenol:chloroform extraction and ethanol precipitation methods, and was resuspended in 100 μL elution buffer (buffer EB, QIAgen, 19086). DNA was further purified and concentrated by Zymo Clean and Concentrator Kit (ZymoResearch, D4014) and eluted with 15 μL buffer EB. To select DNA fragments ranging from 250 to 350 bp in size, DNA samples were loaded on 3% Nusieve™ GTG™ Agarose gel (Lonza, 50081), and electrophoresis was run at 100V in 1× TBE buffer for ∼50 min. The DNA corresponding to the desired size was excised and purified with ZymoClean Gel DNA Recovery Kit (ZymoResearch, D4007) and finally eluted with 50 μL buffer EB. To pull down the biotin-labeled DNA, the samples were incubated with 5 μL of Dynabeads MyOne Streptavidin C1 beads (Invitrogen, 65001) on a slow rotator at room temperature for 20 min. The DNA-bound streptavidin beads were washed twice with 200 μL TWB (5 mM Tris-HCl pH 7.5, 1 mM EDTA, 1 M NaCl, 0.05% Tween 20) and once with 200 μL buffer EB, followed by on-beads library preparation using the NEBNext Ultra II DNA Library Prep Kit for Illumina according to the manufacturer instruction from the end repair to adaptor ligation step. After adapter ligation step, beads were washed twice with 200 μL TWB and once with 200 μL buffer EB, resuspended into 15 μL buffer EB, then processed to PCR amplification step following the protocol from NEBNext Ultra II DNA Library Prep Kit for Illumina. Finally, the DNA library was cleaned twice using AMPure XP Reagent (Beckman Coulter, A63881) and eluted to 25 μL buffer EB. Libraries were sequenced by HiSeqX to generate ∼100 millions of 150-pb paired-end reads per sample.

#### Experimental replication

Some ChIP-seq and Micro-C experiments were biologically duplicated. They include Scc2 ChIP-seq in WT and Δ*wpl1* Δ*eco1*, and Micro-C in WT, Δ*wpl1* Δ*eco1* and Δ*wpl1* Δ*eco1 SCC2-AID* (Figure S3; Tables S2 and S3). After confirming a high similarity between the replicates, one of the datasets was used for detailed downstream analysis and data visualization.

### Quantification and statistical analysis

#### Calibrated ChIP-seq data analysis

Raw sequencing reads were mapped to *S. cerevisiae* and *C. glabrata* reference genomes by Bowtie^58^ using default parameters. For a specific ChIP sample, the number of non-redundant reads mapped to *S. cerevisiae* and *C. glabrata* genome in the input and ChIP fractions were used to calculate the Occupancy Ratio (ORs) using the following formula as described previously.^57^

$$OR=\frac{{ChIP}_{Scer}\times {input}_{Cgla}}{{input}_{Scer}\times {ChIP}_{Cgla}}$$

where ChIP_Scer_, the number of the reads that were in the ChIP sample and mapped to *S. cerevisiae* genome; input_Scer_, the number of the reads that were in the input sample and mapped to *S. cerevisiae* genome; ChIP_Cgla_, the number of the reads that were in the ChIP sample and mapped to *C. glabrata* genome; input_Cgla_, the number of the reads that were in the input sample and mapped to *C. glabrata* genome.

Then, to enable quantitative comparison among multiple ChIP experiments, the normalization factor (NF) of each sample was calculated as described in.^76^

$$NF=\frac{{OR}_{treated}}{{OR}_{control}}$$

where OR_treated_, the OR for the target condition; OR_control_, the OR for the reference condition.

The non-redundant reads mapped to *S. cerevisiae* genome were subject to binning using parse2wig in the DROMPAplus package (version 1.17.2),^59^ and the result was output as a file in wig format. For a ChIP sample, the following normalization option was applied; ‘-n GR –nrpm X’, where X is the product of 1,500,000 and NF for the experiment. Finally, the wig files for input and ChIP samples of a specific ChIP experiment were fed to the PC_ENRICH function in DROMPAplus to generate a file showing normalized ChIP/Input fold-enrichment ratio for each 100-bp genome bin in bigwig or bedgraph format, which was used for downstream analyses. Aggregation plots and heatmaps were produced using deepTools.^60^ To visualize ChIP-seq profiles, Integrative Genomics Viewer^61^ was used. The ChIP-seq peak regions, where the ChIP/Input fold-enrichment ratio was equal to or above the threshold of 2.0, were identified by DROMPA’s PC_ENRICH function. The statistics of ChIP-seq sequencing are in Table S2.

#### Micro-C data analysis

• Contact matrix generation

Sequencing reads were processed to generate a visualizable contact matrix, following the previously described analysis pipeline,^77^ which utilizes bwa for mapping^78^ and pairtools^66^ for processing sequencing reads. For comparison between samples, the valid read pairs of different samples were normalized to the same total read number before generating contact matrices. Juicer Tools^62^ was used to convert.pairs file into a.hic contact matrix that stored the mapped pairs in 5 resolutions (100bp, 200bp, 500bp, 1kb, and 2kp) and 4 normalizations (VC, VC_SQRT, KR, and SCALE), which is ready to be visualized by the compatible web-based software Juicebox (https://www.aidenlab.org/juicebox/). The statistics of Micro-C sequencing is in Table S3.

• Count-versus-distance decaying curve

Iterative correction^79^ was applied to the contact matrix at the resolution of 500 bp using HiCExplorer’s hicCorrect module. The resultant IC-corrected matrix was subjected to HiCExplorer’s hicPlotDistVsCount module^63^ to calculate and produce a result matrix of the number of reads at every 500-bp bin size distance. The output matrix was then used to plot the log scale decaying curve and its first derivative by R.^68^

• Loop calling

For loop calling, .hic files were subjected to the Juicer HICCUPS algorithm^62^ with the following parameters: “-k KR -r 1000 -f 0.1 -p 8 -i 16 -d 2500”, which scanned the contact matrix (normalized by Knight-Ruiz (KR) balancing^80^ and stored in the .hic files at 1kb resolution) using a 16×16-pixel window to search for enriched pixels of peak-width 8. The enriched pixels separated by a distance less than 2500 bp were merged and then filtered by a false discovery rate of 0.1 to generate the final loops list in a bedpe-format file.

• Aggregate peak analysis

The aggregate peak analysis (APA) to present the average contact enrichment score around a set of genome loci (on-diagonal APA) or between pairs of specific loci (off-diagonal APA) was conducted using the Coolpup.py package.^64^ To standardize the contact level with the background, we first calculated the expected interactions for each chromosome arm and then computed the observed-over-expected (observed/expected) enrichment score. The 200-bp resolution contact matrix in .cool format was balanced to filter out poorly mapped bins by *cooler balance*.^65^ The expected interaction was calculated from the balanced contact matrix using the *expected_cis* API module of the cooltools package.^67^ Then, we fed the following three inputs into *Coolpup.py* to calculate and plot the average observed/expected score between pairs of specific sites: the balanced contact matrix, the expected interaction matrix, and the list of the genome loci or paired loci to be analyzed in bedpe format. The list of paired loci of which the averaged pile-up was computed was either a loop file produced by HICCUPS^62^ or a manually generated bedpe file containing, for example, a collection of pairs between a centromeric anchor and a cohesin-bound anchor on chromosome arms grouped by distances between two anchors (used in Figure 5B). *coolpup.py* was executed with the parameters “–flank 10000 –mindist 5000”, which will calculate the average observed/expected score of interactions between the designated pairs of loci separated by a distance larger than 5 kb, in a 20×20-kb window centered at the enriched pixels representing the interaction between two loci. This execution generated a matrix data, which was subsequently used to plot the pile-up heatmap by the *plotpup.py* command.

• Insulation score

The KR-balanced contact matrix at the resolution of 1 kb was subjected to the hicFindTADs module of the HiCExplorer.^63^ The parameters used were “--minDepth 5000 --maxDepth 10000 --step 5000 --correctForMultipleTesting fdr --thresholdComparisons 0.01 --delta 0.01”, which will compute the insulation score on the 1 kb contact matrix at window sizes of 5000 and 10000 using the false discovery rate (FDR) for multiple comparisons with the q-value threshold set to 0.01. The output files consist of lists of boundaries and domains in bed format and genome-wide insulation score in bedGraph format, which can be used to plot the average profile.

#### Statistical test

The two-sided Mann-Whitney *U* test or Student’s *t*-test was used to test whether the difference between two groups is statistically significant.
