# Supplementary material for: Regulation of pericentromeric DNA loop size via Scc2-cohesin interaction
Source: iScience. 2025 Mar 30;28(5):112322. doi: 10.1016/j.isci.2025.112322 (PMC12017868; doi:10.1016/j.isci.2025.112322)
Supplement: Document S1. Figures S1–S17 and Tables S1–S3 [file mmc1.pdf]

iScience, Volume *28*

Supplemental information

Regulation of pericentromeric DNA loop size via Scc2-cohesin interaction

Sao Anh Nguyen, Toyonori Sakata, Katsuhiko Shirahige, and Takashi Sutani

Figure S1

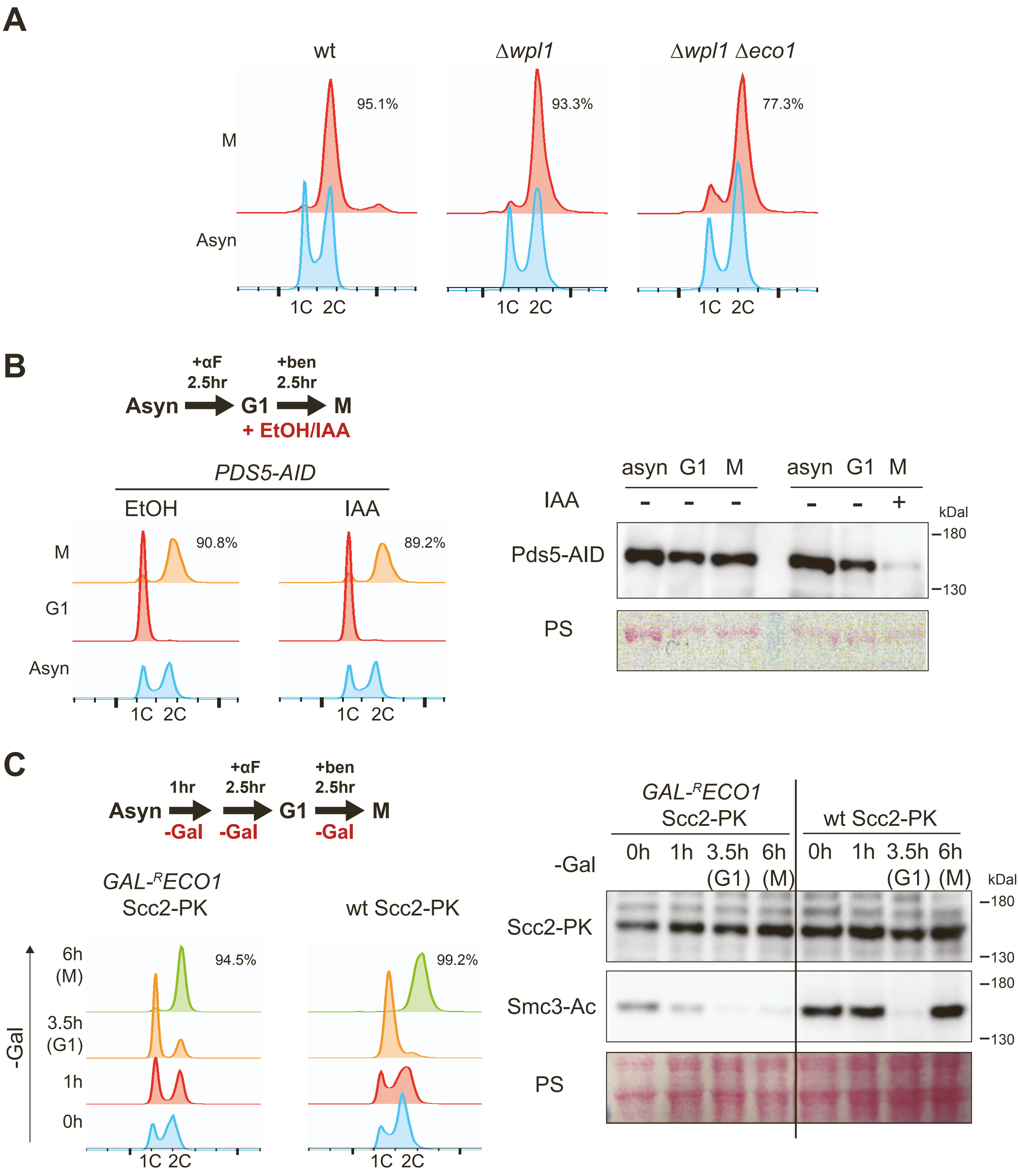
A
wt
Δwpl1
Δwpl1 Δeco1
M
Asyn
95.1%
93.3%
77.3%
1C 2C
B
+αF
2.5hr
+ben
2.5hr
Asyn
G1
M
+ EtOH/IAA
PDS5-AID
EtOH
IAA
90.8%
89.2%
asyn G1 M asyn G1 M
IAA
- - - - - +
kDal
Pds5-AID
PS
180
130
C
1hr
-Gal
+αF
2.5hr
+ben
2.5hr
GAL-RECO1
Scc2-PK
wt Scc2-PK
6h
(M)
3.5h
(G1)
1h
0h
94.5%
99.2%
-Gal
0h 1h 3.5h 6h
(G1) (M)
Smc3-Ac

Figure S1. Validation of cell cycle arrest and protein depletion. Related to Figure 1 and 2.
(A) Cell cycle arrest monitoring by flow cytometry. Asyn, asynchronous; M, metaphase. The proportion of cells with 2C DNA content was indicated. **(B)** (Left) Cell culture condition and cell cycle arrest monitoring by flow cytometry. To deplete Pds5 protein in metaphase-arrested cells, *PDS5-AID* cells arrested in G1 phase by α-factor (αF) were released into the medium containing benomyl (Ben) and idole-3-acetic acid (IAA) and cultured for 2.5 h. The proportion of cells with 2C DNA content was indicated. (Right) Western blot assessing IAA-dependent degradation of Pds5-AID. PS, Ponceau S staining as a loading control. **(C)** (Left) Cell culture condition and cell cycle arrest monitoring by flow cytometry. To prepare the cells that proceeded through S phase without Eco1 protein and arrested in metaphase, *GAL-^{R}ECO1* cells grown in galactose-containing medium were transferred to galactose-free medium (YPD) and cultured for 1 h at 23°C, followed by the addition of 2 μM α-factor and cultivation for additional 2.5 h. Then, the cells were released into α-factor-free YPD containing benomyl and cultured for 2.5 h. The proportion of cells with 2C DNA content was indicated.. Gal, galactose. (Right) Western blot to verify the depletion of Eco1 in a galactose-free medium. Eco1 depletion resulted in the disappearance of acetylated cohesin (Smc3-Ac) in metaphase.

Figure S2

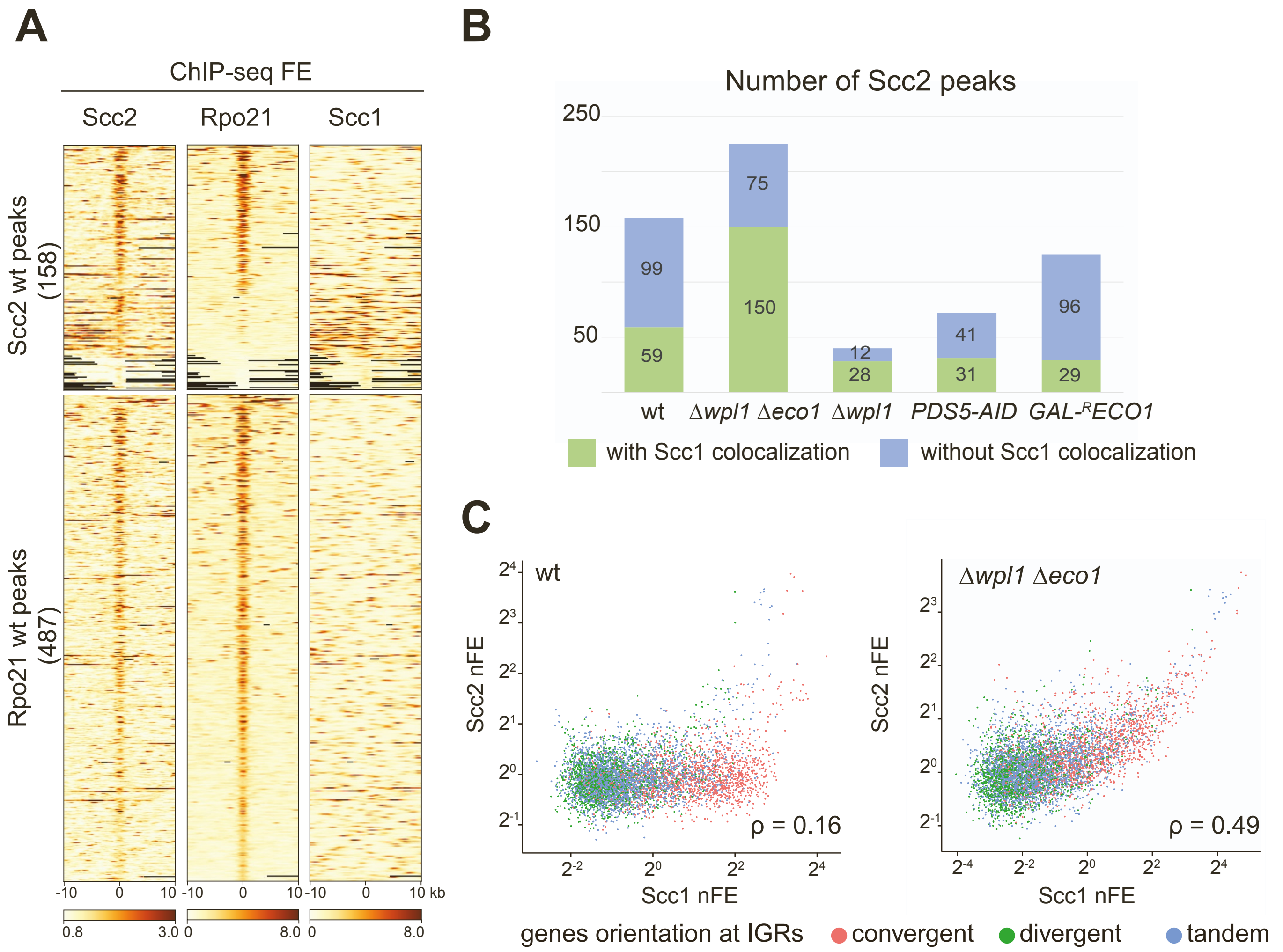

Figure S2. Colocalization of Scc2 with RNA pol II in wt and with cohesin in Δ*wpl1* Δ*eco1*. Related to Figure 1 and 2.

(A) Heatmap of Scc2, Rpo21 (the largest subunit of RNA pol II), and Scc1 ChIP-seq FE in wt. 10-kb surrounding regions of Scc2 and Rpo21 peaks in wt are depicted. Regions are sorted in descending order of Rpo21 FE in wt. **(B)** The number of Scc2 peaks in the genome of the indicated strains. Green, those overlapping with the Scc1 peaks; blue, those not overlapping with the Scc1 peaks. **(C)** Correlation between Scc1 and Scc2 ChIP-seq nFE in wt and Δ*wpl1* Δ*eco1*. Each dot corresponds to an intergenic region (IGR). The color of the dot indicates the orientation of the genes adjacent to the IGR. ρ, Spearman's correlation coefficient.

Figure S3

A

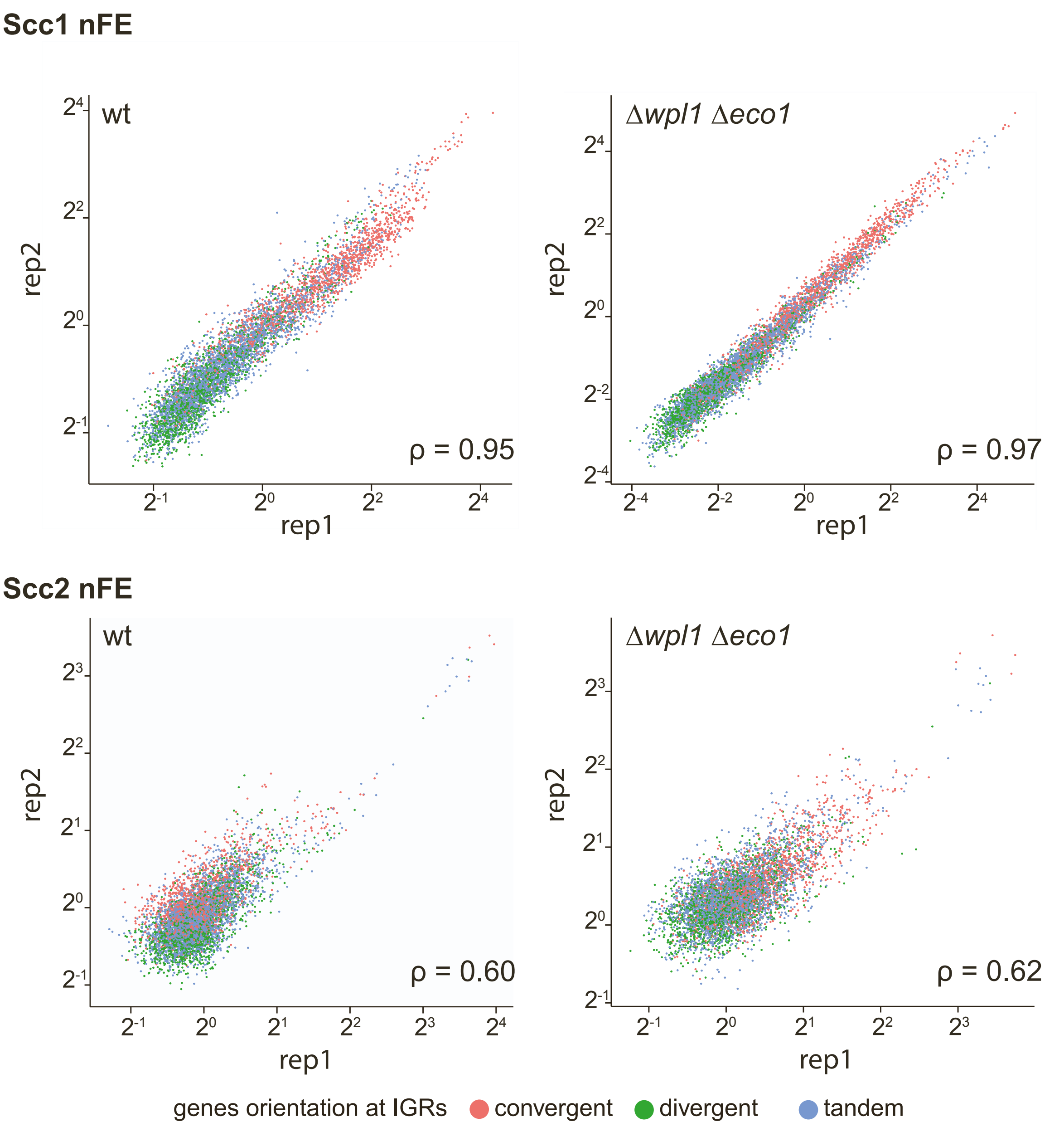
Scc1 nFE
wt
Δwpl1 Δeco1
rep2
rep1
ρ = 0.95
ρ = 0.97
Scc2 nFE
wt
Δwpl1 Δeco1
rep2
rep1
ρ = 0.60
ρ = 0.62
genes orientation at IGRs
convergent
divergent
tandem

B

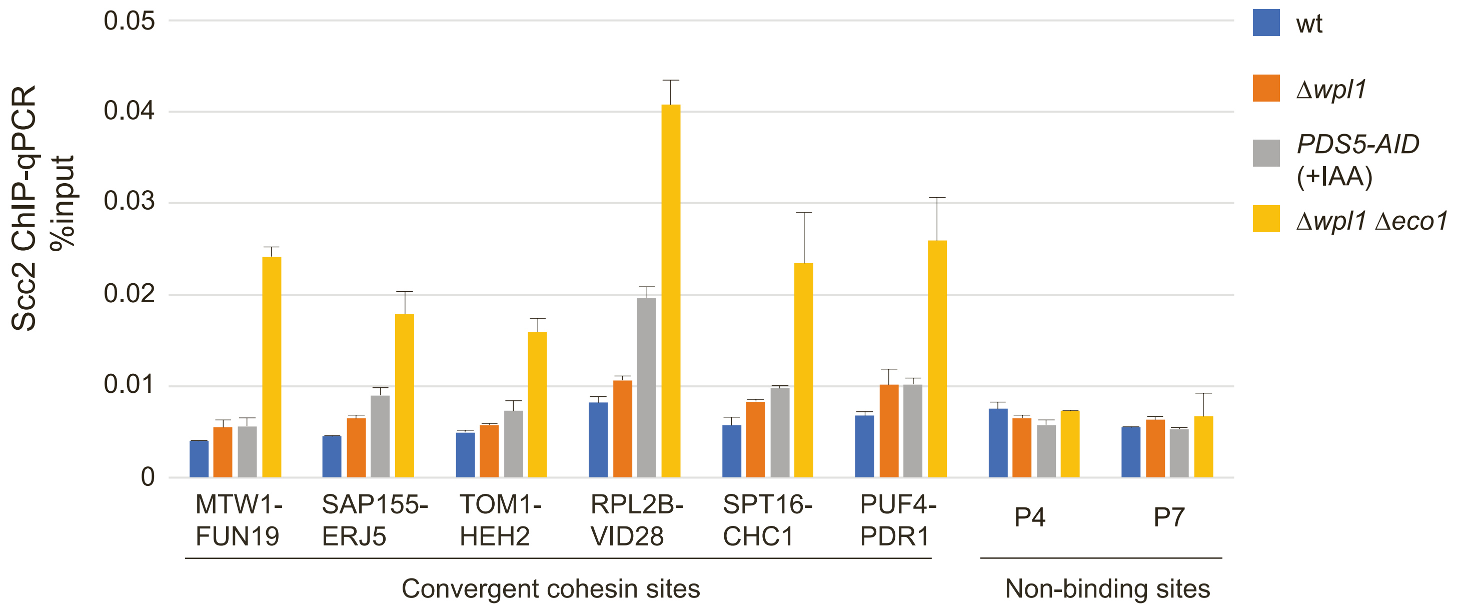
Scc2 ChIP-qPCR
%input
0.05
0.04
0.03
0.02
0.01
0
wt
Δwpl1
PDS5-AID (+IAA)
Δwpl1 Δeco1
MTW1-FUN19
SAP155-ERJ5
TOM1-HEH2
RPL2B-VID28
SPT16-CHC1
PUF4-PDR1
P4
P7
Convergent cohesin sites
Non-binding sites

Figure S3. Reproducibility and validation of ChIP-seq data. Related to Figure 1 and 2.
(A) Correlation between two biological replicates of Scc1 or Scc2 ChIP-seq. nFE values at all intergenic regions (IGRs) are depicted. Results in wt and Δ*wpl1* Δ*eco1* are shown. The color of the dot indicates the orientation of the genes adjacent to the IGR. ρ, Spearman's correlation coefficient. **(B)** ChIP-qPCR of Scc2 in wt, Δ*wpl1, PDS5-AID* (+IAA) and Δ*wpl1* Δ*eco1*. Analyzed loci are six representative cohesin binding sites and two negative control sites (non-binding sites). See Table S2 for detailed information on the primers used. The mean of two technical replicates was shown. Error bar, standard deviation.

Figure S4

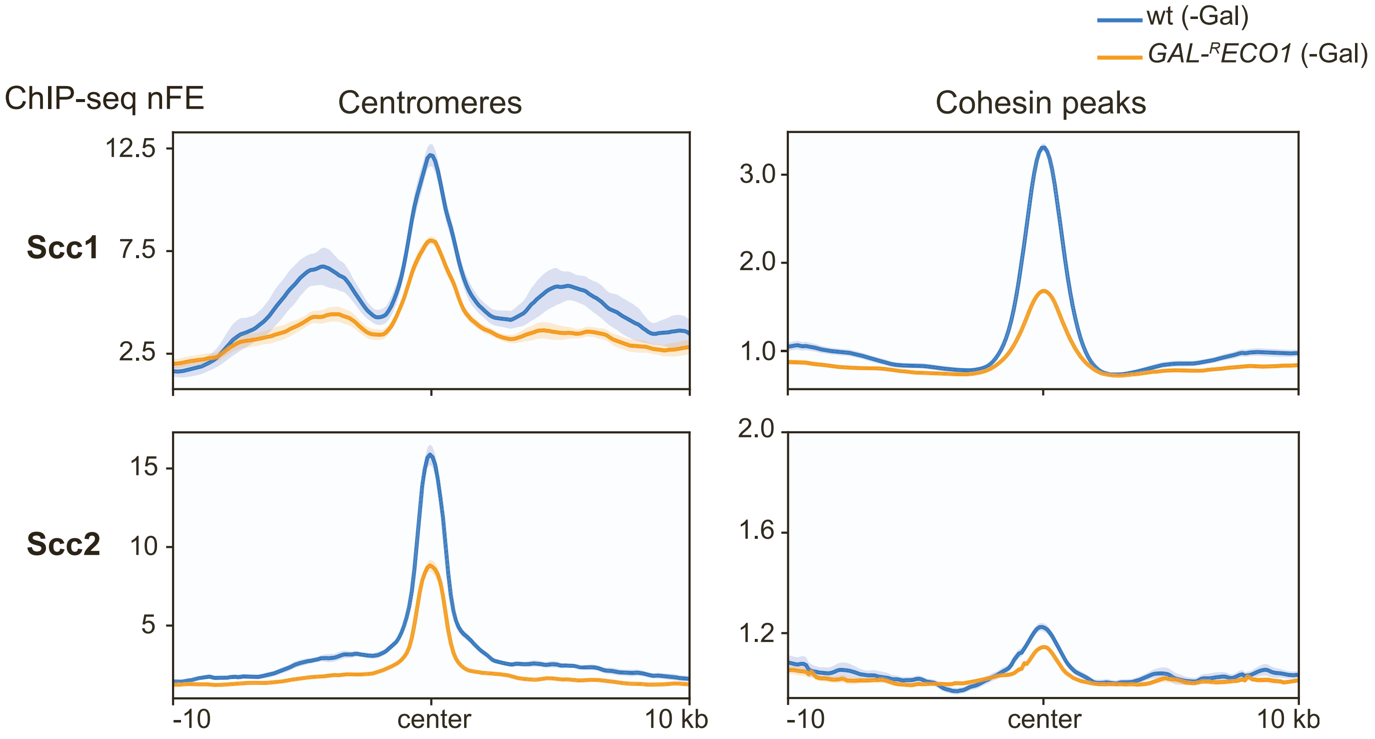

Figure S4. Scc1 and Scc2 chromosomal binding in *GAL-^{R}ECO1*. Related to Figure 1-2.
Aggregated ChIP-seq profiles of Scc1 and Scc2 in wt and *GAL-^{R}ECO1* cultured in galactose-free medium. Wt and *GAL-^{R}ECO1* strains with *SCC1*-PK gene (SN27 and SN39, respectively), and wt and *GAL-^{R}ECO1* strains with *SCC2*-PK gene (SN40 and SN41, respectively) were used. The profiles are centered at the centromeres or the summit of non-centromeric Scc1 peaks in wt. Bold line, mean; shaded area, 95% confidence interval.

Figure S5

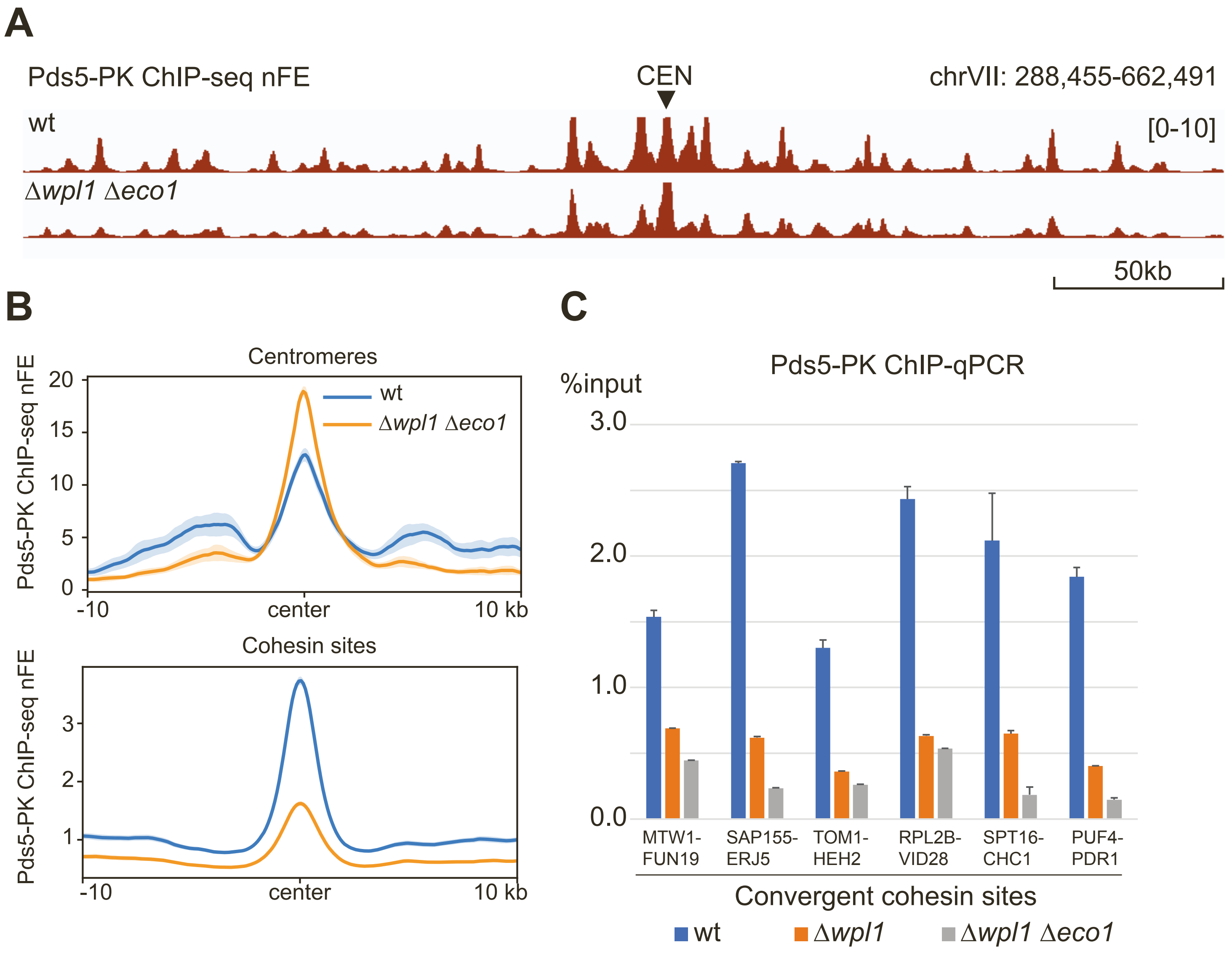

Figure S5. Impaired Pds5 co-localization at the cohesin sites in Δ*wpl1* Δ*eco1*. Related to Figure 1 and 2. **(A)** Calibrated ChIP-seq profiles of Pds5-PK in wild-type (SN47) and Δ*wpl1* Δ*eco1* (SN48). Cells are arrested at metaphase by benomyl treatment. **(B)** Aggregated ChIP-seq profiles of Pds5 in wt and Δ*wpl1* Δ*eco1*. The profiles are centered at the centromeres or the summit of non-centromeric Scc1 peaks in wt. Bold line, mean; shaded area, 95% confi-dence interval. **(C)** ChIP-qPCR of Pds5 in wt, Δ*wpl1* (SN49), and Δ*wpl1* Δ*eco1*. Analyzed loci are six representative cohesin binding sites. The mean of two technical replications was shown. Error bar, standard deviation.

Figure S6

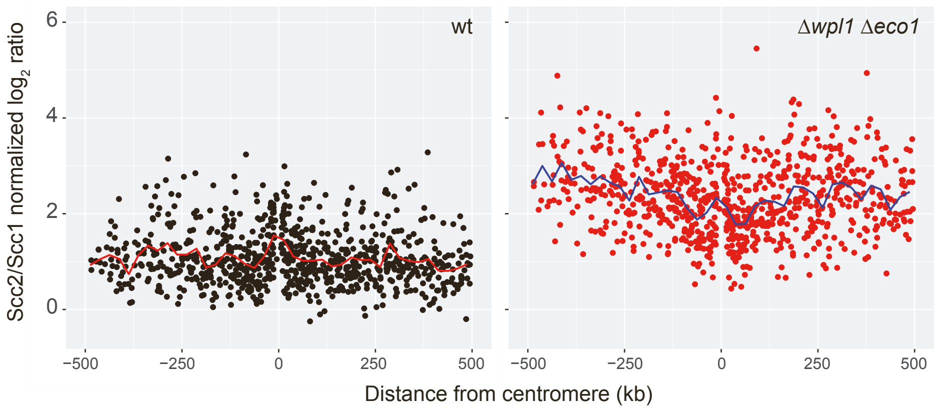

Figure S6. Correlation between Scc2-cohesin interaction and genomic location. Related to Figure 1 and 2.
Scatter plots showing the relationship between the ratio of Scc2 nFE to Scc1 nFE (in log2) and the distance from the centromere. Each dot represents a cohesin binding site. The line indicates the average of each 2.5-kb bin.

Figure S7

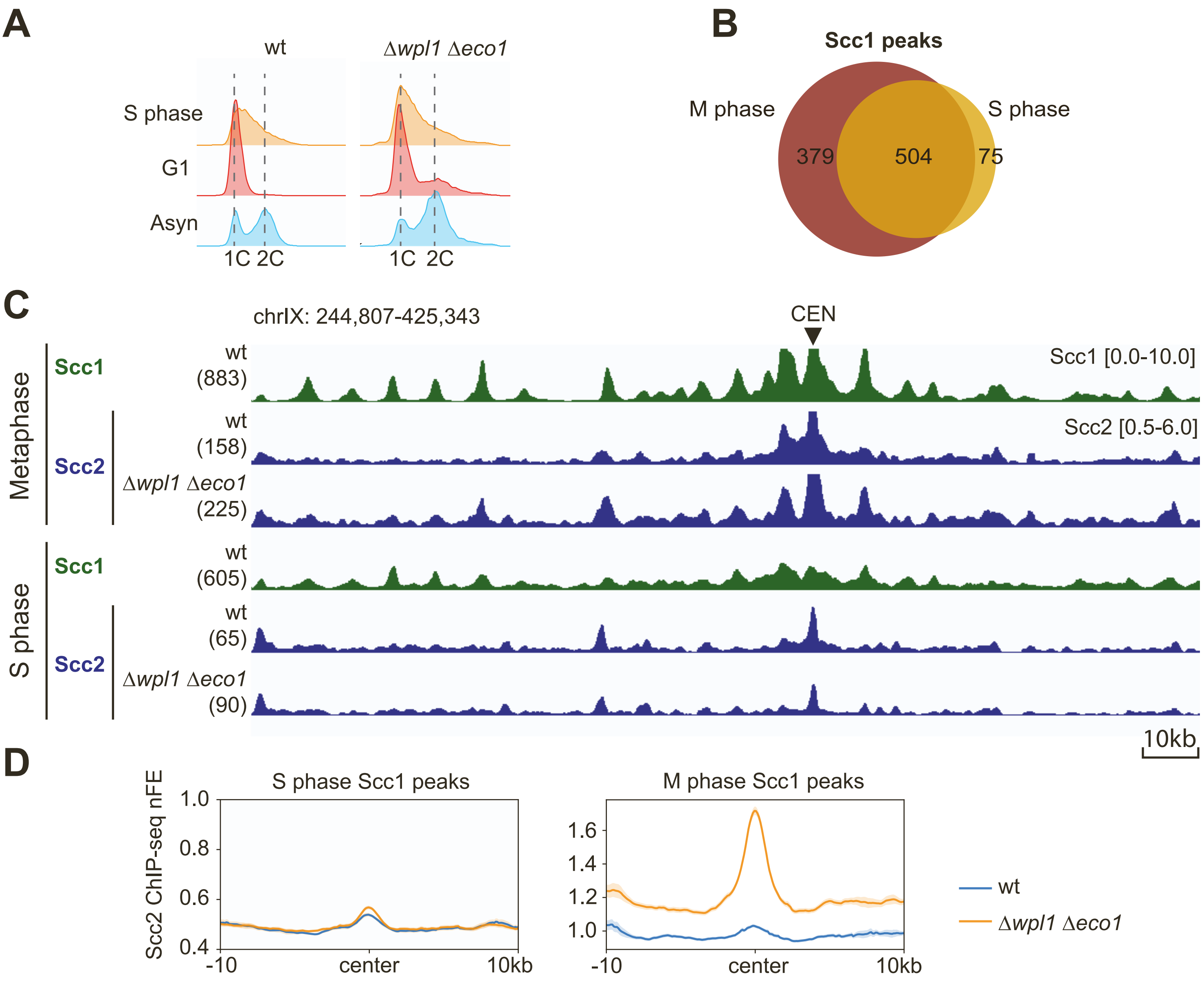

Figure S7. Scc2 colocalization with cohesin in Δ*wpl1* Δ*eco1* is specific to metaphase. Related to Figure 1 and 2.
(A) Cell cycle monitoring by flow cytometry. Asyn, asynchronous. Cells were synchronized in S phase by releasing G1-arrested cells for 30 mins in α-factor-free medium. **(B)** Venn diagram illustraing the overlap of Scc1 peaks in S phase and M phase. **(C)** Calibrated ChIP-seq profiles of Scc1 and Scc2 in the indicated conditions. **(D)** Aggregated ChIP-seq profiles of Scc2 in S- and M-phase at the region of +/- 10kb centered at all non-centromeric cohesin sites. Bold line, mean; shaded area, 95% confidence interval. Note that the y-axis scales are different between the two plots.

Figure S8

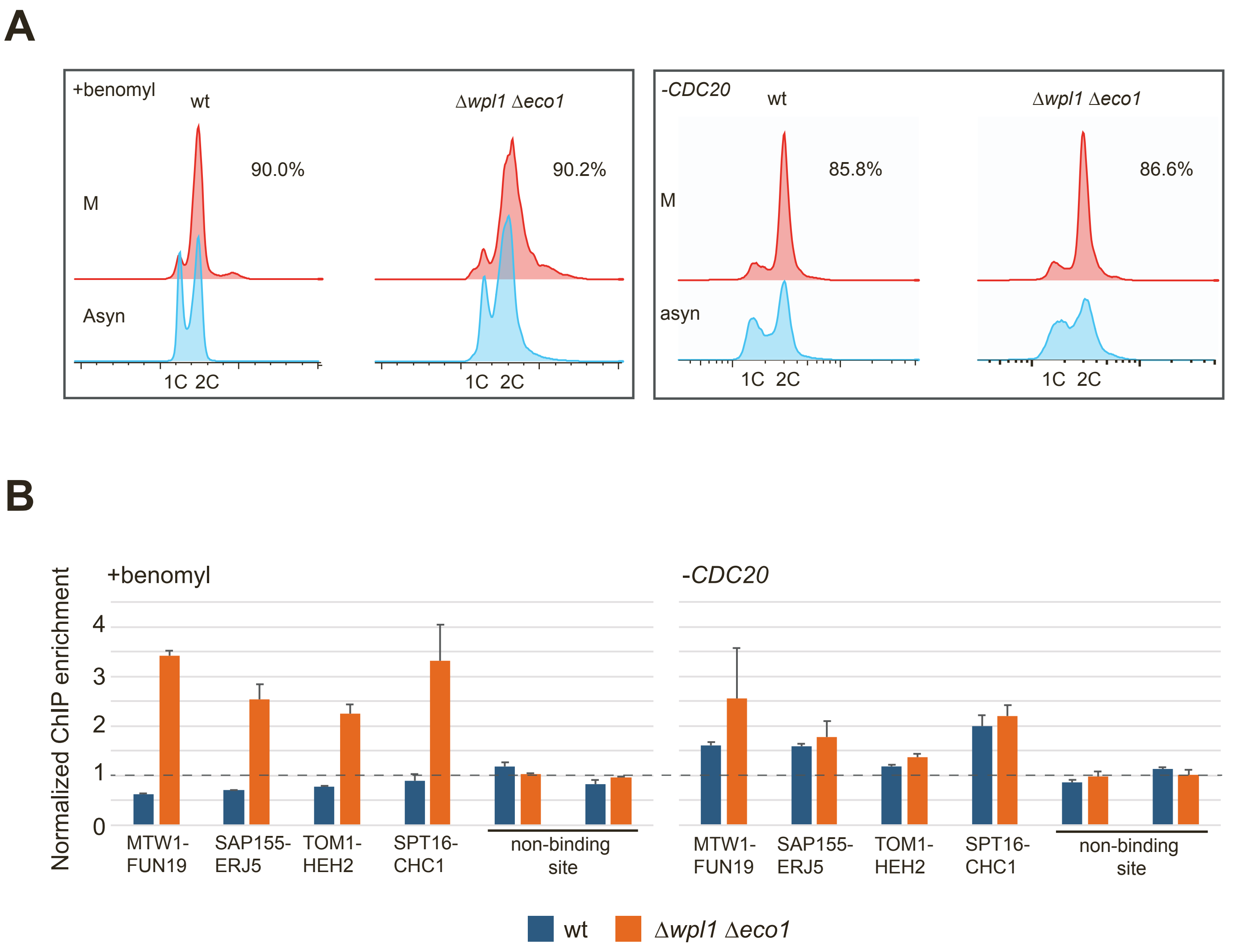

Figure S8. Scc2 chromosomal binding in Δ*wpl1* Δ*eco1* cells arrested in metaphase by Cdc20 depletion. Related to Figure 1 and 2.

(A) Cell cycle arrest monitored by flow cytometry. Metaphase arrest was induced by benomyl treatment or Cdc20 depletion. Cdc20 depletion was accomplished by culturing asynchronous wt (TS732) and Δ*wpl1* Δ*eco1* (TS733) strains with *GAL-CDC20* gene in galactose-free YPD medium for 2.5 h. Asyn, asynchronous; M, metaphase. The proportion of cells with 2C DNA content was indicated. **(B)** ChIP-qPCR of Scc2-PK at four cohesin sites and two non-binding sites in wt and Δ*wpl1* Δ*eco1* arrested at metaphase by either benomyl or Cdc20 depletion. The ChIP enrichment values were normalized by setting the average enrichment of the non-binding sites to 1. The data for benomyl-treated cells is identical to that shown in Figure S3B.

Figure S9

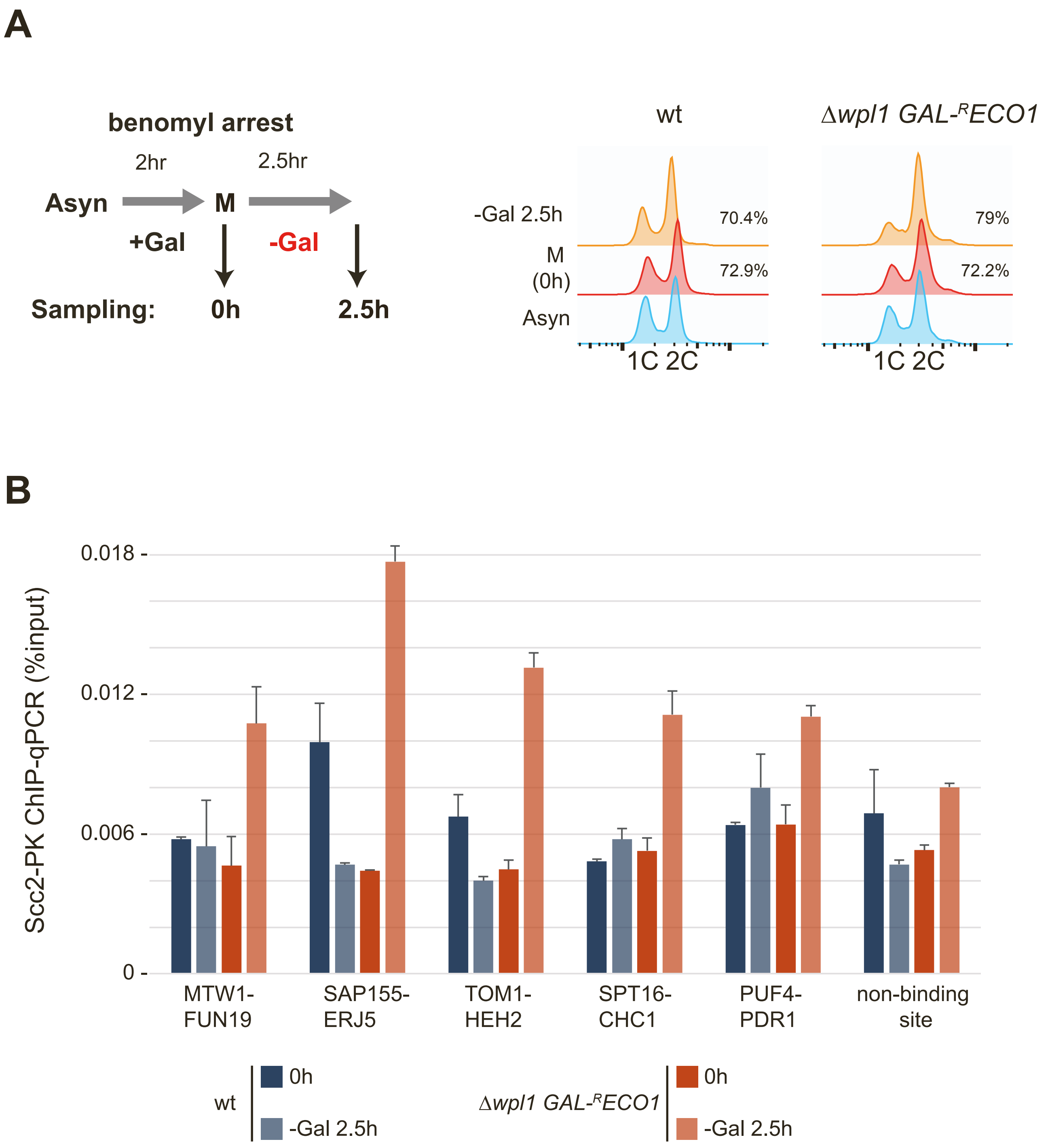

Figure S9. Replicated experiment supporting Figure 2C
(A) Cell culture condition and cell cycle arrest monitoring by flow cytometry. Eco1 was depleted by culturing metaphase-arrested *GAL-^{R}ECO1* cells in a galactose-free YPD medium for 2.5 h. Samples were taken before and after Eco1 repression and subject to Scc2 ChIP-qPCR. Asyn, asynchronous; M, metaphase; Gal, galactose. The proportion of cells with 2C DNA content was indicated. **(B)** ChIP-qPCR of Scc2-PK in the indicated conditions. Analyzed loci are four representative cohesin binding sites and two non-binding sites. The mean of two technical replicates was shown. Error bar, standard deviation.

Figure S10

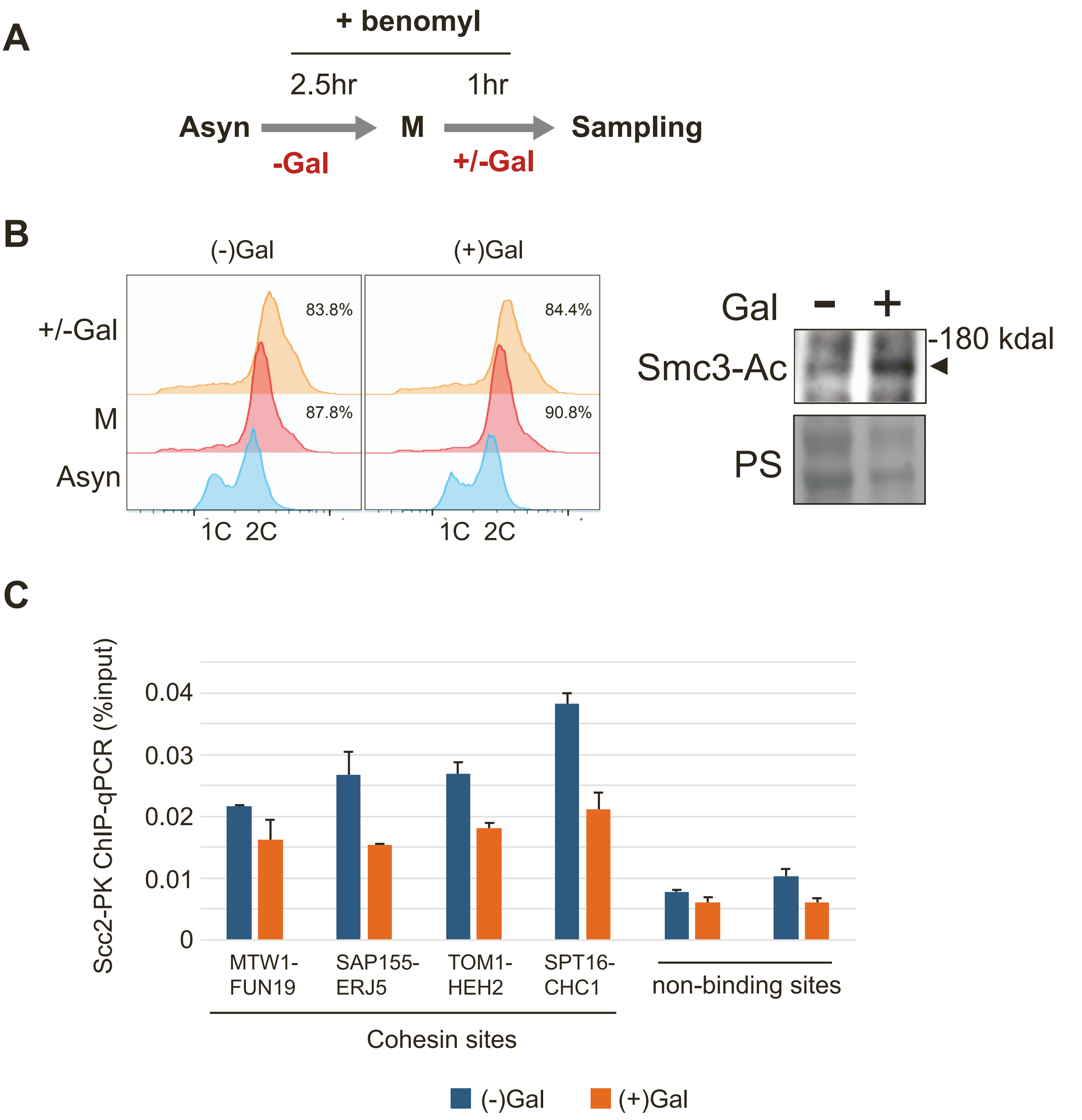

A
+ benomyl
2.5hr
1hr
Asyn
M
Sampling
-Gal
+/-Gal
B
(-)Gal
(+)Gal
+/-Gal
M
Asyn
83.8%
87.8%
84.4%
90.8%
1C 2C
1C 2C
Gal
-
+
-180 kdal
Smc3-Ac
PS
C
Scc2-PK ChIP-qPCR (%input)
0.04
0.03
0.02
0.01
0
MTW1-FUN19
SAP155-ERJ5
TOM1-HEH2
SPT16-CHC1
non-binding sites
Cohesin sites
(-)Gal
(+)Gal

Figure S10. Effect of mitotic Eco1 induction on Scc2 chromosomal binding. Related to Figure 2.

(A) Schematic representation of the experimental protocol to induce Eco1 expression in Δ*wpl1* cells (SN722) arrested in metaphase. **(B)** Cell cycle arrest monitored by flow cytometry (left) and western blot to check the Smc3 acetylation level (right). The proportion of cells with 2C DNA content was indicated in the flow cytometry data. Black triangle in the western blot image indicates the position of acetylated Smc3**.** Asyn, asynchronous; M, metaphase; Gal, galactose; Smc3-Ac, acetylated Smc3; PS, ponceau S staining as a loading control. **(C)** ChIP-qPCR of Scc2-PK in the indicated conditions. Analyzed loci are four representative cohesin binding sites and two non-binding sites. The mean of two technical replications was shown. Error bar, standard deviation.

Figure S11

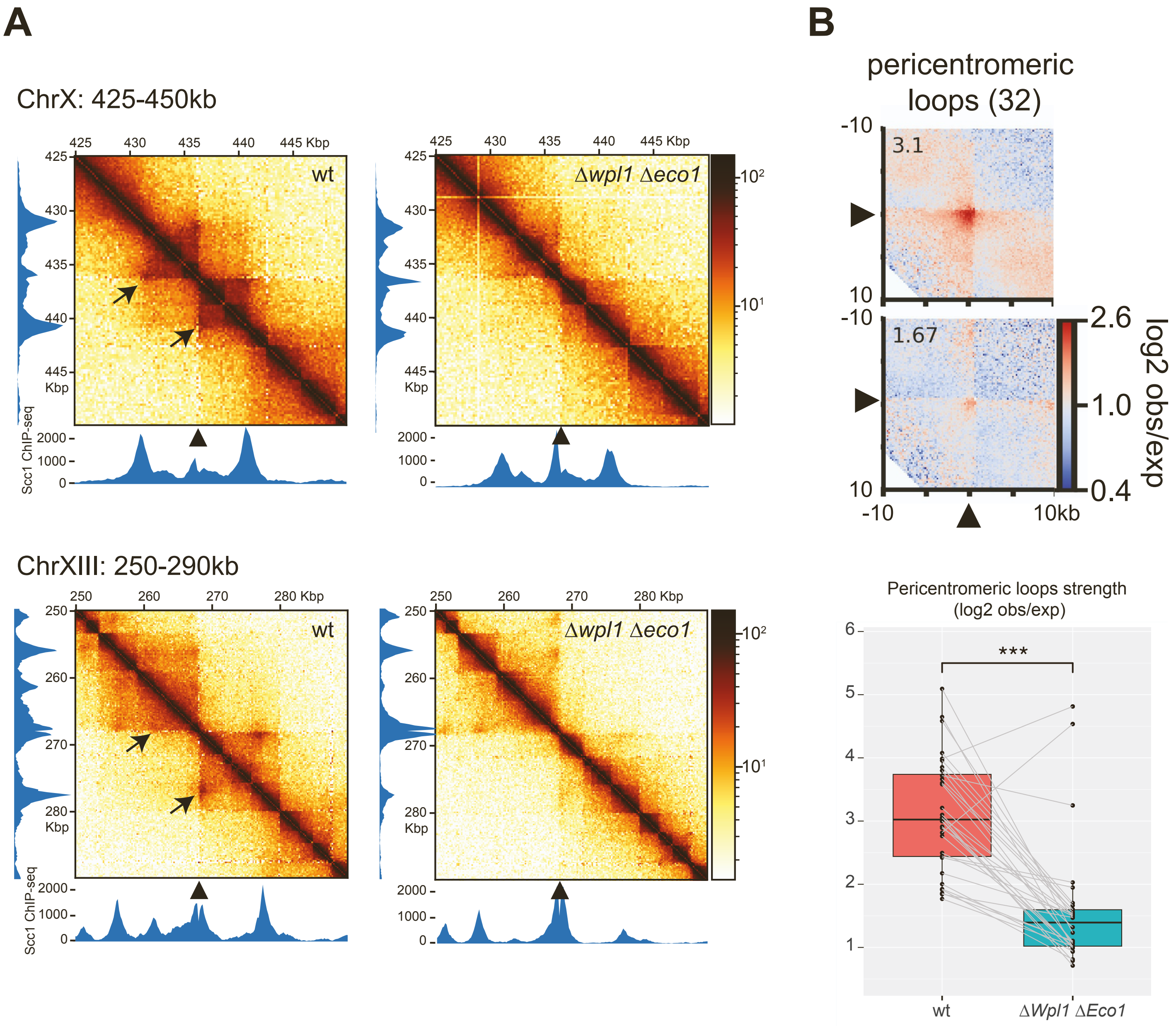

Figure S11. Pericentromeric loops in wt and Δ*wpl1* Δ*eco1*. Related to Figure 3.
(A) Zoomed-in view of the contact maps shown in Figure 3A. The pericentromeric regions were magnified and visualized. Scc1 ChIP-seq profile is displayed on the left side and at the bottom of the contact map. Black arrows, pericentromeric loop; black triangles, centromeres. Contact maps were computed on 200 bp-resolution data. **(B)** Average contact frequency (represented as observed/expected ratio) between a centromere and centromere-proximal anchor sites observed in wt, and paired boxplot to show the difference of the contact frequency between wt and Δ*wpl1* Δ*eco1*. The number in the top-left corner of each plot indicates the average enrichment score of the 3 × 3 central pixels. Two-tailed t-test was conducted to evaluate the statistical significance of the difference. ***, $p < 0.001$.

Figure S12

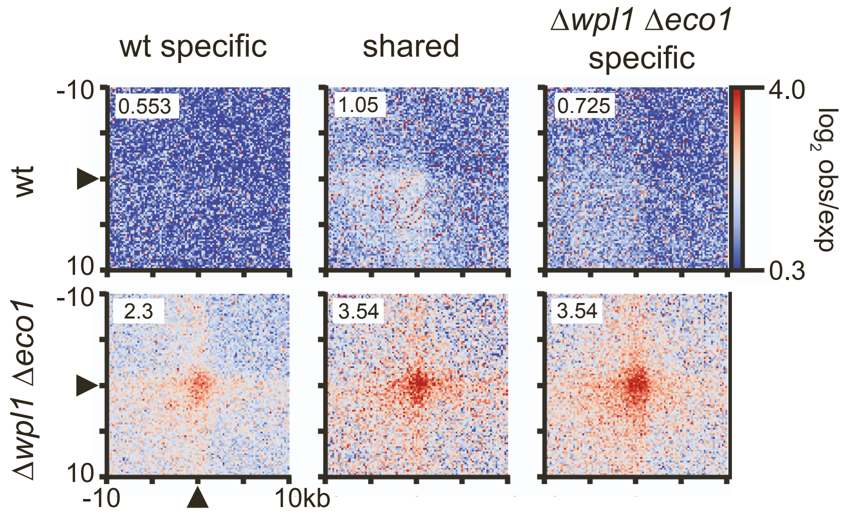

Figure S12. Interaction between centromeres and wt-specific anchors in Δ*wpl1* Δ*eco1*. Related to Figure 3. Average contact frequency (represented as observed/expected ratio) between centromeres and cohesin-bound anchor sites. The anchor sites were divided into three groups: wt-specific, Δ*wpl1* Δ*eco1*-specific, and shared anchors (as in Figure 3G). Black triangles indicate the two anchor loci of each pair. The number in the top-left corner of each plot indicates the average enrichment score of the 3 x 3 central pixels.

Figure S13

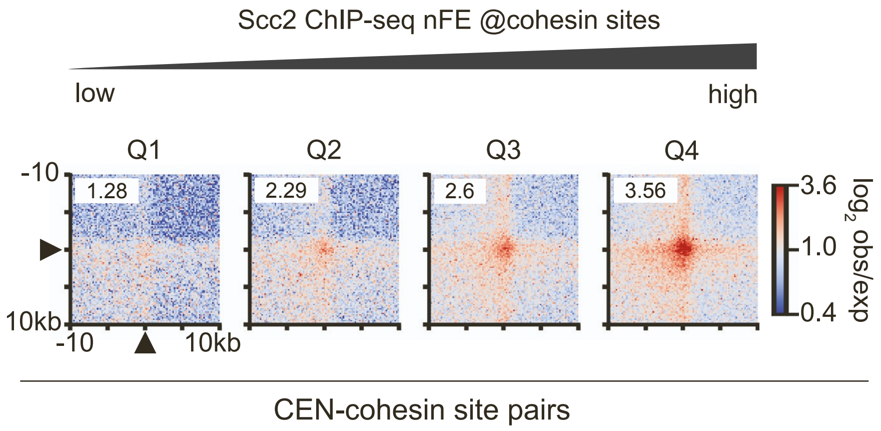

Figure S13. Correlation between Scc2 chromosomal binding and contact frequency of the centromere-originated DNA loops. Related to Figure 4.

Average contact frequency (represented as observed/expected ratio) between a centromere and a cohesin site on the same chromosome in Δ*wpl1* Δ*eco1*. The centromere-cohesin site pairs were divided into four groups according to the Scc2 nFE value at the cohesin site (Q1 to Q4 in order of increasing FE), as described in Figure 1C. Black triangles indicate the two anchor loci of each pair. The number in the top-left corner of each plot indicates the average enrichment score of the 3 x 3 central pixels.

Figure S14

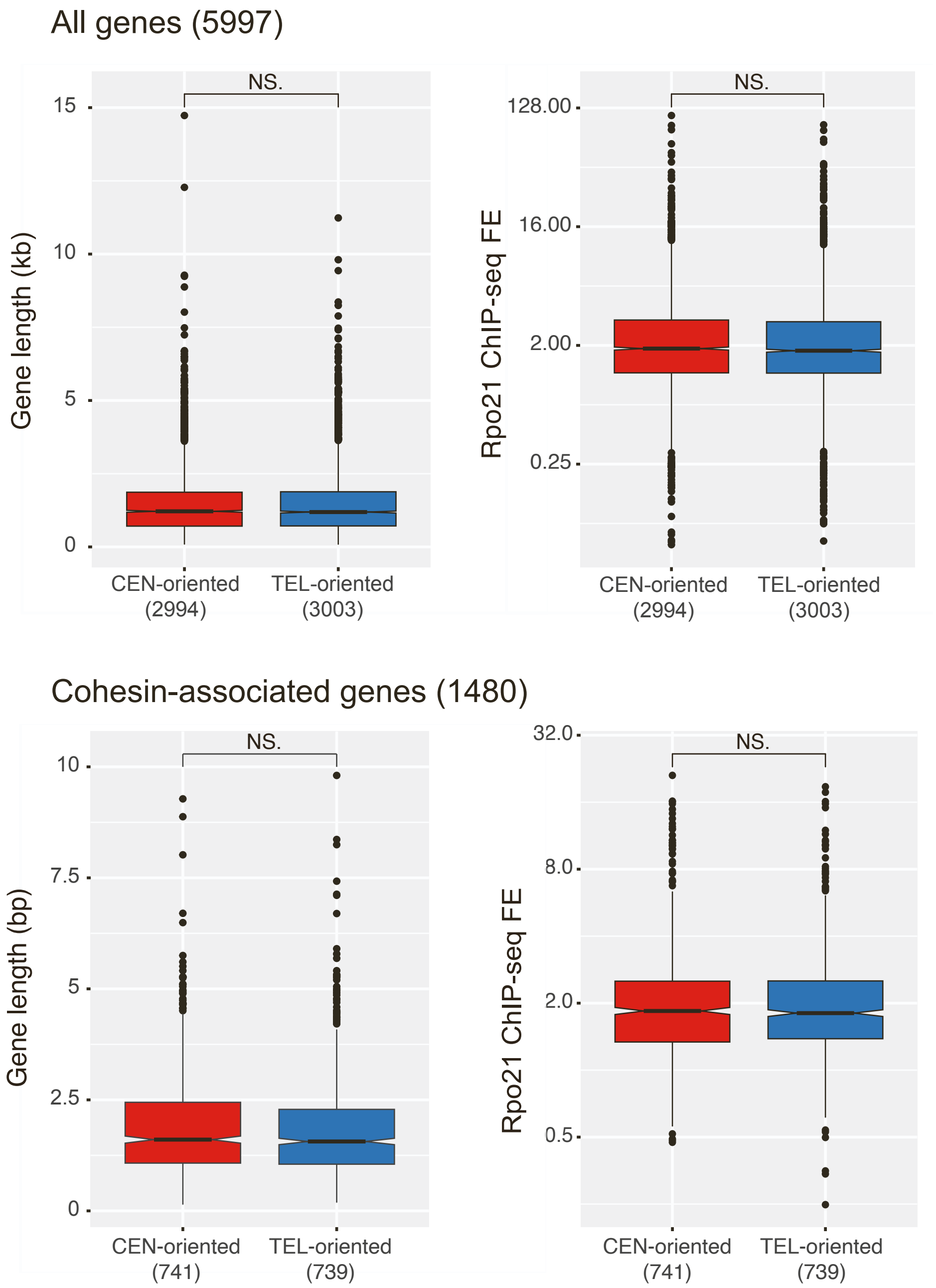

Figure S14. Control analysis for Figure 4D
The gene length and Rpo21 binding level were compared between (i) CEN- and TEL-oriented genes in the genome (All genes) and (ii) CEN- and TEL-oriented genes adjacent to the cohesin binding sites (cohesin associated genes). Two-tailed Mann-Whitney U test was conducted to evaluate the statistical significance of the difference. NS, non-significant difference ($p > 0.05$).

Figure S15

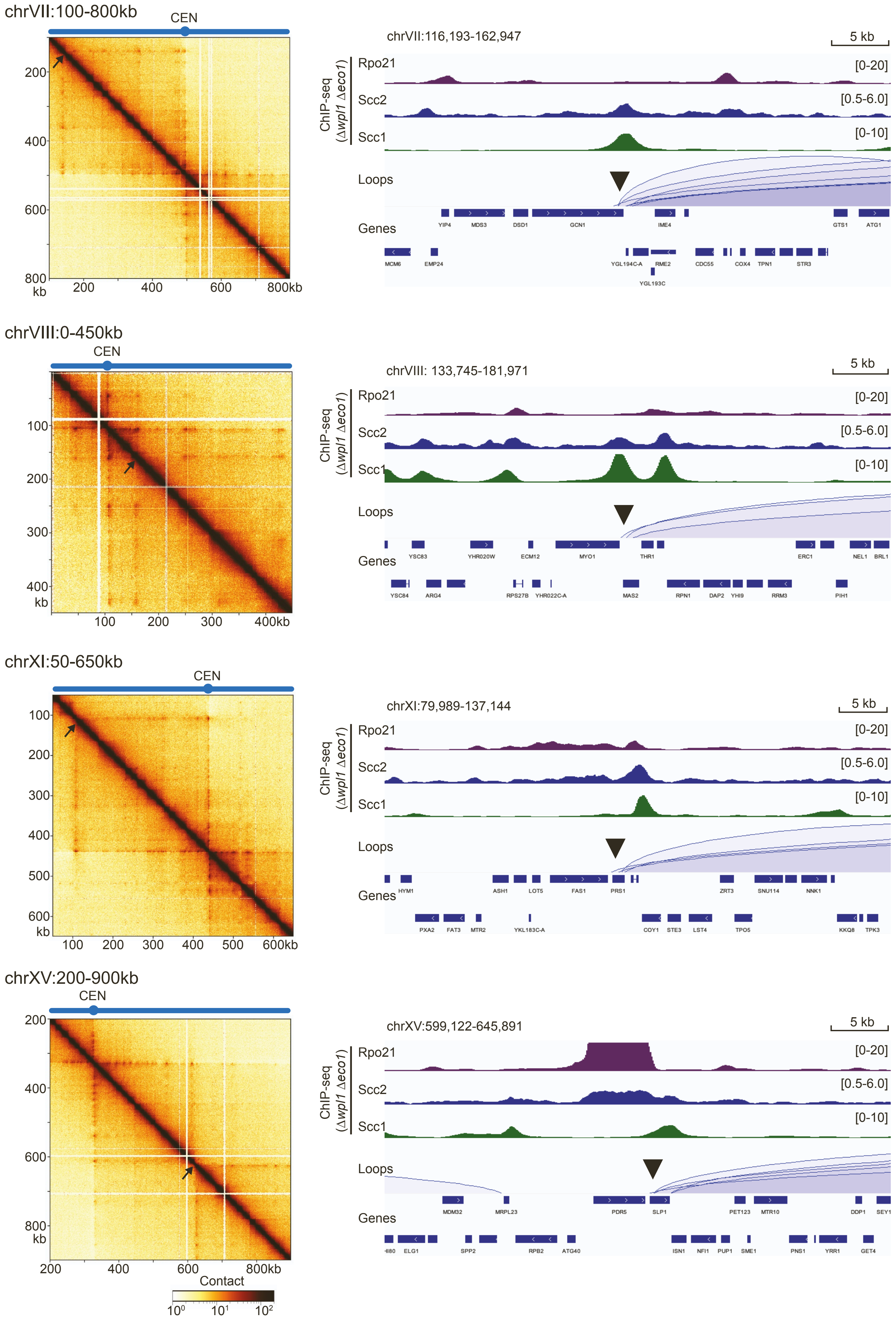

chrVII:100-800kb
CEN
200
400
600
800
kb
200
400
600
800kb
chrVII:116,193-162,947
5 kb
ChIP-seq
(Δwpl1 Δeco1)
Rpo21
[0-20]
Scc2
[0.5-6.0]
Scc1
[0-10]
Loops
Genes
YIP4
MDS3
DSD1
GCN1
IME4
GTS1
ATG1
MCM6
EMP24
YGL194C-A
RME2
CDC55
COX4
TPN1
STR3
YGL193C
chrVIII:0-450kb
CEN
100
200
300
400
kb
100
200
300
400kb
chrVIII: 133,745-181,971
5 kb
ChIP-seq
(Δwpl1 Δeco1)
Rpo21
[0-20]
Scc2
[0.5-6.0]
Scc1
[0-10]
Loops
Genes
YSC83
YHR020W
ECM12
MYO1
THR1
ERC1
NEL1
BRL1
YSC84
ARG4
RPS27B
YHR022C-A
MAS2
RPN1
DAP2
YHI9
RRM3
PIH1
chrXI:50-650kb
CEN
100
200
300
400
500
600
kb
100
200
300
400
500
600kb
chrXI:79,989-137,144
5 kb
ChIP-seq
(Δwpl1 Δeco1)
Rpo21
[0-20]
Scc2
[0.5-6.0]
Scc1
[0-10]
Loops
Genes
HYM1
ASH1
LOT5
FAS1
PRS1
ZRT3
SNU114
NNK1
PXA2
FAT3
MTR2
YKL183C-A
COY1
STE3
LST4
TPO5
KKQ8
TPK3
chrXV:200-900kb
CEN
200
400
600
800
kb
200
400
600
800kb
Contact
10^0
10^1
10^2
chrXV:599,122-645,891
5 kb
ChIP-seq
(Δwpl1 Δeco1)
Rpo21
[0-20]
Scc2
[0.5-6.0]
Scc1
[0-10]
Loops
Genes
MDM32
MRPL23
PDR5
SLP1
PET123
MTR10
DDP1
SEY1
ELG1
SPP2
RPB2
ATG40
ISN1
NFI1
PUP1
SME1
PNS1
YRR1
GET4

Figure S15. Potential strong barrier sites for loop extrusion found in chromosome arms of *Δwpl1 Δeco1*. Related to Figure 4.

(Left) Micro-C contact map at 1-kb resolution in the indicated genome regions. Potential strong barrier sites for loop extrusion are marked with black arrows. CEN, centromere. (Right) ChIP-seq profiles of Rpo21, Scc2, and Scc1 in Δ*wpl1* Δ*eco1* around the potential barrier sites (black arrowheads). The detected DNA loops (Loops), as well as the genes annotated in the RefSeq (Genes), are also shown.

Figure S16

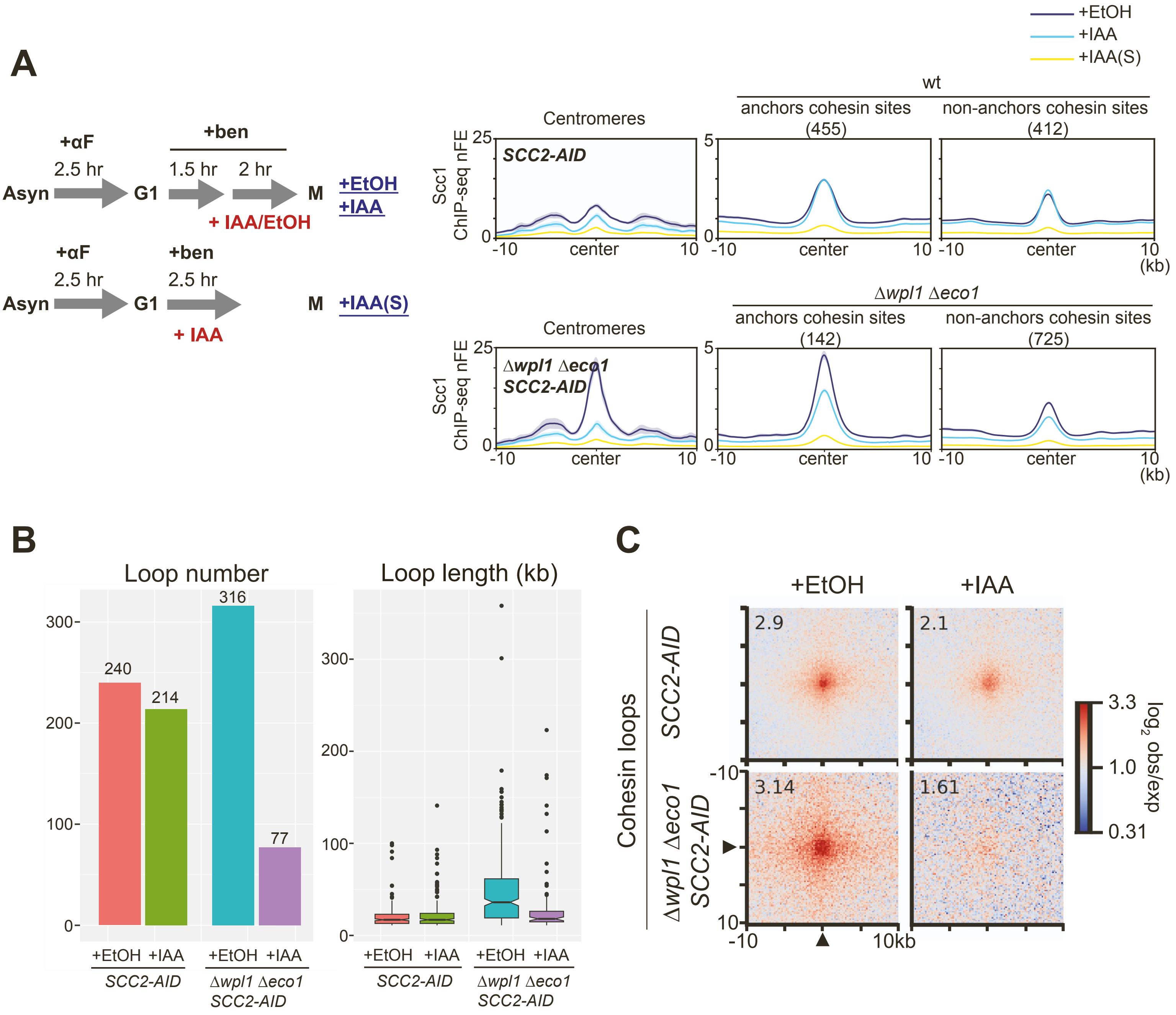

Figure S16. Validation of culture condition and features of DNA loops in Scc2-depleted cells. Related to Figure 6.

(A) (Left) Schematic representation of the experimental protocol used to arrest cells in metaphase and induce rapid degradation of Scc2-AID. The cells were arrested in metaphase before Scc2 depletion in the '+IAA' condition while the cells proceeded through S phase without Scc2 in the '+IAA(S)' condition. (Right) Aggregated plots of Scc1 ChIP-seq nFE in *SCC2-AID* and Δ*wpl1* Δ*eco1 SCC2-AID* strains cultured under the indicated conditions (+EtOH, +IAA, +IAA(S)). 10-kb surrounding regions of the centromeres, anchor cohesin sites, and non-anchor cohesin sites are averaged and depicted. Bold line, mean; shaded area, 95% confidence interval. **(B)** Comparison of the number and length of the DNA loops detected in the indicated samples. **(C)** Average contact frequency of the cohesin loops (loops connecting between cohesin binding sites) detected in vehicle-treated *SCC2-AID* and Δ*wpl1* Δ*eco1 SCC2-AID* (+EtOH). The averaged contact frequency for the same locus-pair in IAA-treated condition was shown side by side for comparison (+IAA).

Figure S17

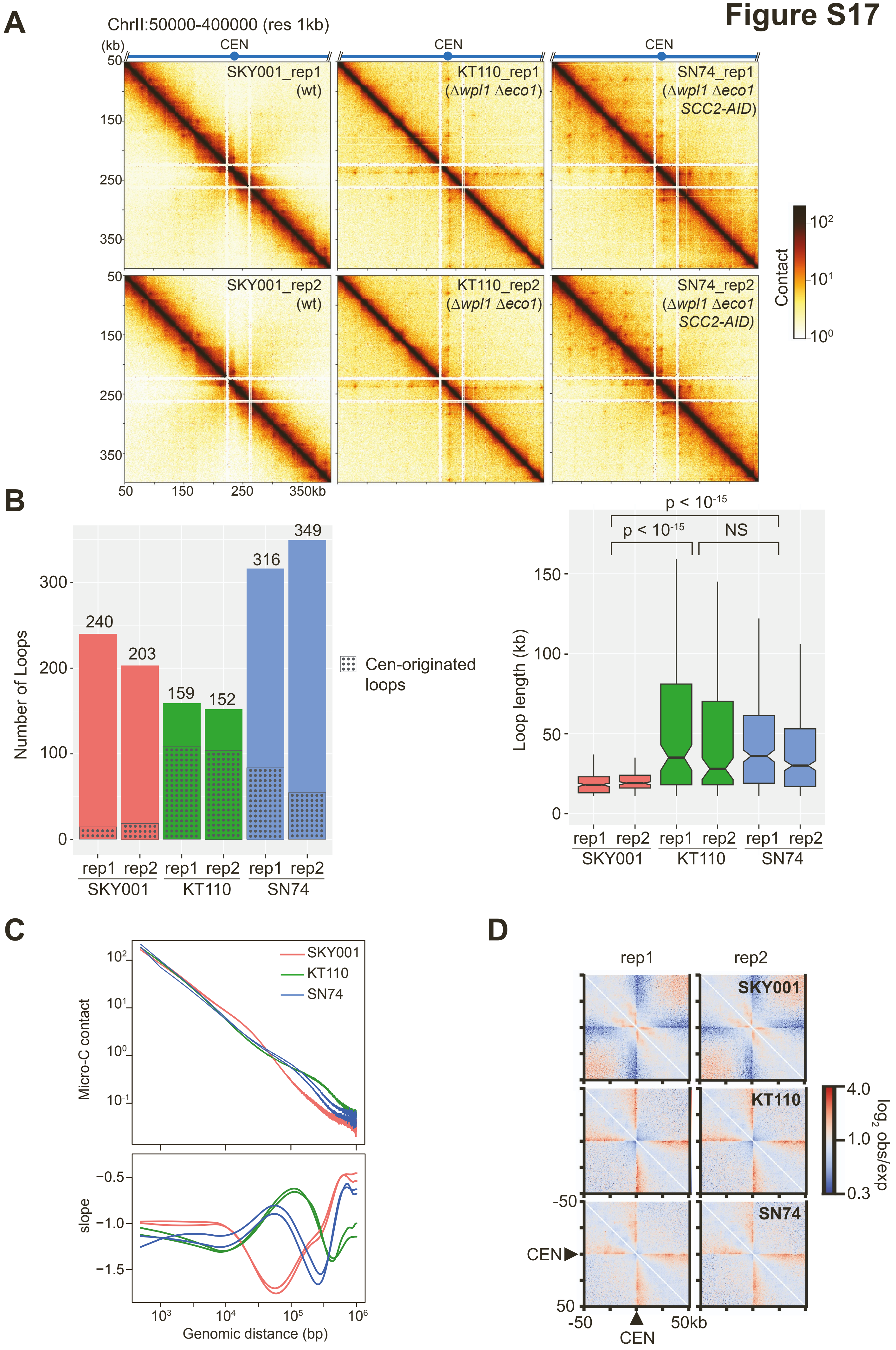
A
ChrII:50000-400000 (res 1kb)
(kb)
CEN
SKY001_rep1 (wt)
KT110_rep1 (Δwpl1 Δeco1)
SN74_rep1 (Δwpl1 Δeco1 SCC2-AID)
SKY001_rep2 (wt)
KT110_rep2 (Δwpl1 Δeco1)
SN74_rep2 (Δwpl1 Δeco1 SCC2-AID)
Contact
50 150 250 350kb
B
Number of Loops
240
203
159
152
316
349
Cen-originated loops
rep1 rep2 rep1 rep2 rep1 rep2
SKY001 KT110 SN74
p < 10-15
p < 10-15
NS
Loop length (kb)
C
SKY001
KT110
SN74
Micro-C contact
slope
Genomic distance (bp)
D
rep1
rep2
SKY001
KT110
SN74
log2 obs/exp
4.0
1.0
0.3
CEN
-50
50
50kb

Figure S17. Micro-C data reproducibility and minor difference between strains. Related to Figure 6.
(A) Contact matrix at the resolution of 1 kb of SKY001 (wt), KT110 (Δ*wpl1* Δ*eco1*), and SN74 (Δ*wpl1* Δ*eco1* *SCC2-AID SCC1-9PK*). For each strain, two biological replicates are shown. The number of valid reads in each sample was normalized to 42 million. **(B)** The number and length of pericentromeric loops detected in the indicated samples. The outliers were not depicted in the box plot. Data from the two replicates were shown. The numbers above the plots are p-values (Mann-Whitney U test, two-tailed). NS, non-significant difference ($p > 0.05$). **(C)** Contact-versus-distance decaying curves of IC-corrected contact matrix and their first derivatives (slope), at the resolution of 500 bp. Results of two biological replicates for SKY001, KT110, and SN74 strains are shown. **(D)** Average contact frequency around the centromeres in the indicated samples. Black triangles indicate the centromere position.

Supplement Table S1. Primer pairs used for ChIP-qPCR. Related to STAR Methods.

ID	Forward	Reverse	Target
**P4**	CACCGTGCTCCAAATGGCCT	AGCTTCGCTGCTTGATGCGT	chrII: 541262-541355
**P7**	GCGGCCGAACGGTCACTAGA	TGAGCCAATGCTTCCAGAGGCTA	chrII: 569861-569922
**P11**	AGGAACGTCCTTCGAATCCCTGA	ACGCTAACTCCACGTCGTTCACT	chrI: 79982-80107 (MTW1-FUN19)
**P12**	AGTCGACTTGGATGCCGCAA	AGGGTCTTCAGGACGAGATTTCAA	chrVI: 216353-216624 (SAP155-ERJ5)
**P15**	AAACCATATTGAAACAGCGAATGGT	TGGAGAGATCATGACATGTTGGG	chrVI: 1379871-1380069 (TOM1-HEH2)
**P16**	ACTACCTTTTGCAGCCGGGGT	TCTGGAACCAGGGCATTCACCA	chrIX: 318368-318569 (RPL2B-VID28)
**P17**	GTCGACTTGGATGCCGCAAT	AGGGTCTTCAGGACGAGATTTCA	chrVI: 216300-216700 (MET10-SMC2)
**P18**	TCCACGTCTTAAAATCCTGTGGGT	CCGAAGAACTCGCTAAAAAGTCCG	chrVII: 102535-102623 (SPT16-CHC1)
**P20**	AGAGAAGGAGATGCCCTAGAAAACA	AGTATCCTGTGGAGCGACGTT	chrVII: 468980-469107 (PUF4-PDR1)

Supplemental Table S2. ChIP-seq sequencing statistics. Related to STAR Methods.

Sample	Total reads	Scer		Cgla		ORs	NFs	# used for read normalization	Relevant figures
		Uniquely mapped reads	%	Uniquely mapped reads	%				
Scc2-PK wt IP_rep1	6472695	2452200	37.88530125	2954642	45.64778659	0.097863022	1	1500000	Fig. S3
Scc2-PK wt WCE_rep1	7144449	5415436	75.79921139	638559	8.937834114				
Scc2-PK Δeco1 Δwpl1 IP_rep1	5013750	2117407	42.23200199	1460546	29.13081027	0.12430746	1.270218895	1905329	Fig. S3
Scc2-PK Δeco1 Δwpl1 WCE_rep1	6798302	4863347	71.5376722	417007	6.133987575				
Scc2-PK wt IP_rep2	6392707	518654	8.113214011	5290250	82.75445754	0.066295358	1	1500000	Fig. 1, 2, 3, 4, S2, S3, S4, S6, S7
Scc2-PK wt WCE_rep2	7441356	4014324	53.94613562	2714526	36.47891594				
Scc2-PK Δeco1 Δwpl1 IP_rep2	12267808	944791	7.70138398	10649111	86.80532822	0.071064364	1.071935745	1607904	Fig. 1, 2, 3, 4, S2, S3, S4, S6, S7, S15
Scc2-PK Δeco1 Δwpl1 WCE_rep2	13558489	7513487	55.41537114	6018261	44.38740187				
Scc2-PK Δwpl1 IP	4909748	989811	20.1601182	2872937	58.51495841	0.055980069	0.84440405	1266607	Fig. 1, 2, S2
Scc2-PK Δwpl1 WCE	6972689	5325310	76.37383512	865271	12.40943057				
Scc2-PK wt_S IP	2898231	1183230	40.82593831	1305152	45.03271133	0.027656719	0.417174294	625762	Fig. S7
Scc2-PK wt_S WCE	4158198	3497417	84.10895777	106694	2.565871082				
Scc2-PK Δeco1 Δwpl1_S IP	2748831	980774	35.6796762	1340074	48.75068711	0.026799413	0.404242678	606365	Fig. S7
Scc2-PK Δeco1 Δwpl1_S WCE	4141942	2990835	72.20851958	109516	2.644073722				
Scc2-PK Pds5-AID_EtOH IP	4911028	2983248	60.74589679	1239974	25.24876665	0.061036733	1	1500000	Fig. 1
Scc2-PK Pds5-AID_EtOH WCE	5799281	5314736	91.6447401	134833	2.324995116				
Scc2-PK Pds5-AID_IAA IP	6147883	340848	5.544152353	5105647	83.04723756	0.05513342	0.903282615	1354924	Fig. 1, S2
Scc2-PK Pds5-AID_IAA WCE	6550227	3082080	47.0530258	2545358	38.85908076				
Scc2-PK wt_Glu IP	2158161	573276	19.42885633	1584885	50.55966631	0.180878384	1	1500000	Fig. 1, 2
Scc2-PK wt_Glu WCE	4063358	2708800	58.52585472	1354558	28.5462418				
Scc2-PK gal-ub-eco1_Glu IP	4526737	2913293	64.35746101	1150940	25.42537815	0.131435076	0.726648881	1089974	Fig 1, 2, S2
Scc2-PK gal-ub-eco1_Glu WCE	5497945	4953896	90.10450268	257233	4.678711773				
Pds5-PK wt IP	3880013	2943690	75.86804477	254119	6.549436819	5.37904526	1	1500000	Fig. 2, S5
Pds5-PK wt WCE	4559051	2811051	61.6586873	1305326	28.6315288				
Pds5-PK Δwpl1 Δeco1 IP	4884452	3351888	68.62362451	860322	17.61348049	3.158911687	0.587262522	880894	Fig. 2, S5
Pds5-PK Δwpl1 Δeco1 WCE	4610114	1993369	43.23903921	1616206	35.05783154				
Scc1-PK wt_Glu IP	5641594	3565032	63.19192767	1477868	26.19592973	1.945643469	1	1500000	Fig. 2
Scc1-PK wt_Glu WCE	6147371	3234123	52.6098555	2608507	42.43288716				
Scc1-PK gal-ub-eco1_Glu IP	3367198	1612993	47.90312301	1004300	29.82598588	1.613099911	0.829082994	1243625	Fig. 2
Scc1-PK gal-ub-eco1_Glu WCE	5740738	2734643	47.63573952	2746584	47.84374413				
Scc1-PK wt IP	10415274	4190427	40.23347826	5237703	50.28867219	1.540681638	1	1500000	Fig. 1, 2, 3, 4, S2, S3, S6, S7
Scc1-PK wt WCE	8239307	2625194	31.86183013	5055416	61.35729619				
Scc1-PK Δeco1 Δwpl1 IP	18067791	8023149	44.40581032	10033074	55.53016415	1.064940649	0.691213955	1036821	Fig. 1, 2, 3, 4, S2, S3, S6, S15
Scc1-PK Δeco1 Δwpl1 WCE	21558985	9245763	42.88589189	12312814	57.11221563				
Scc1-PK wt (Scc2-AID, EtOH) IP	15007440	6986349	46.55256993	7939155	52.90146088	0.893368225	1	1500000	Fig. S3, S16
Scc1-PK wt (Scc2-AID, EtOH) WCE	19388527	9619991	49.61692551	9766280	50.37143874				
Scc1-PK Scc2-AID_G2/M IP	7532484	2497240	33.15294131	3730839	49.52999568	0.661045065	0.739946919	1109921	Fig. S16
Scc1-PK Scc2-AID_G2/M WCE	6215713	2631317	42.33330915	2598666	41.80801141				
Scc1-PK Scc2-AID_S IP	7245745	1346198	18.57915232	4337777	59.86654236	0.248702003	0.278386892	417581	Fig. S16
Scc1-PK Scc2-AID_S WCE	8151344	3881665	47.6199385	3110682	38.16158415				
Scc1-PK Δeco1 Δwpl1 (Scc2-AID, EtOH) IP	15026713	4521746	30.09138459	10443130	69.4971016	0.780452492	0.873606728	1310411	Fig. S3, S16
Scc1-PK Δeco1 Δwpl1 (Scc2-AID, EtOH) WCE	25672631	9160538	35.6821161	16511707	64.31638035				
Scc1-PK Δeco1 Δwpl1 Scc2-AID_G2/M IP	15492620	3979475	25.68626223	11429056	73.77096966	0.600714192	0.672414997	1008623	Fig. S16
Scc1-PK Δeco1 Δwpl1 Scc2-AID_G2/M WCE	19066009	6995702	36.69201037	12069347	63.30295449				
Scc1-PK Δeco1 Δwpl1 Scc2-AID_S IP	8225039	1026696	12.48256695	5921304	71.99119664	0.173864851	0.194617232	291926	Fig. S16
Scc1-PK Δeco1 Δwpl1 Scc2-AID_S WCE	9318949	4337444	46.54434744	4349318	46.67176524				
Rpo21_wt_IP	3744311	3706943	99.00200598	NA	NA	NA	NA	1500000	Fig. S2
Rpo21 Δeco1 Δwpl1_IP	3770581	3709096	98.36934945	NA	NA	NA	NA	1500000	Fig. 4, S14, S15
Rpo21 Δeco1 Δwpl1_WCE	4116966	3807160	92.47489535	NA	NA	NA	NA	1500000	

Supplement Table S3. Micro-C sequencing statistics. Related to STAR Methods.

Sample	Strain	Relevant figures	Valid read pairs	Cis	Trans
wt_rep1	SKY001	Fig. 3, S11, S12, S17	119,690,695	84,759,731	34,930,964
wt_rep2	SKY001	Fig. S17	115,657,691	75,560,544	40,097,147
*Δwpl1 Δeco1*_rep1	KT110	Fig. 3, S11, S12, S13, S15, S17	115,443,402	80,102,530	35,340,872
*Δwpl1 Δeco1*_rep2	KT110	Fig. S17	107,953,022	71,779,182	36,173,840
wt *pds5-aid* (EtOH)	SN80	Fig. 5	86,898,327	61,971,243	24,927,084
wt *pds5-aid* (IAA)	SN80	Fig. 5	109,137,056	74,506,203	34,630,853
Δwpl1	KT127	Fig. 5	124,119,589	72,499,681	51,619,908
Δwpl1 Δeco1 SCC2-PK	SN54	Fig. 5	100,326,853	70,685,184	29,641,669
wt scc2-aid (EtOH)	SN75	Fig. 6,S16	28,410,279	18,623,591	9,786,688
wt scc2-aid (IAA)	SN75	Fig. 6, S16	33,101,775	18,319,770	14,782,005
Δwpl1 Δeco1 scc2-aid (EtOH)_rep1	SN74	Fig. 6, S16, S17	44,163,578	28,617,556	15,546,022
Δwpl1 Δeco1 scc2-aid (EtOH)_rep2	SN74	Fig. 6, S17	83,346,041	57,423,809	25,922,232
Δwpl1 Δeco1 scc2-aid (IAA)_rep1	SN74	Fig. 6, S16, S17	164,153,206	77,743,353	86,409,853
Δwpl1 Δeco1 scc2-aid (IAA)_rep2	SN74	Fig. 6, S17	152,672,879	88,652,111	64,020,768
